# RAG1 Core and V(D)J Recombination Signal Sequences Were Derived from *Transib* Transposons

**DOI:** 10.1371/journal.pbio.0030181

**Published:** 2005-05-24

**Authors:** Vladimir V Kapitonov, Jerzy Jurka

**Affiliations:** **1**Genetic Information Research Institute, Mountain ViewCaliforniaUnited States of America; Scripps Research InstituteUnited States of America

## Abstract

The V(D)J recombination reaction in jawed vertebrates is catalyzed by the RAG1 and RAG2 proteins, which are believed to have emerged approximately 500 million years ago from transposon-encoded proteins. Yet no transposase sequence similar to RAG1 or RAG2 has been found. Here we show that the approximately 600-amino acid “core” region of RAG1 required for its catalytic activity is significantly similar to the transposase encoded by DNA transposons that belong to the *Transib* superfamily. This superfamily was discovered recently based on computational analysis of the fruit fly and African malaria mosquito genomes. *Transib* transposons also are present in the genomes of sea urchin, yellow fever mosquito, silkworm, dog hookworm, hydra, and soybean rust. We demonstrate that recombination signal sequences (RSSs) were derived from terminal inverted repeats of an ancient *Transib* transposon. Furthermore, the critical DDE catalytic triad of RAG1 is shared with the *Transib* transposase as part of conserved motifs. We also studied several divergent proteins encoded by the sea urchin and lancelet genomes that are 25%−30% identical to the RAG1 N-terminal domain and the RAG1 core. Our results provide the first direct evidence linking RAG1 and RSSs to a specific superfamily of DNA transposons and indicate that the V(D)J machinery evolved from transposons. We propose that only the RAG1 core was derived from the *Transib* transposase, whereas the N-terminal domain was assembled from separate proteins of unknown function that may still be active in sea urchin, lancelet, hydra, and starlet sea anemone. We also suggest that the RAG2 protein was not encoded by ancient *Transib* transposons but emerged in jawed vertebrates as a counterpart of RAG1 necessary for the V(D)J recombination reaction.

## Abstract 2

RAG1 and RAG2 activity is central to adaptive immunity, but the precise evolutionary origins of these proteins were unknown; the authors argue that RAG1 evolved from the Transib family of transposons.

## Introduction

The immune system of jawed vertebrates detects and destroys foreign invaders, including bacteria and viruses, by a specific response to an unlimited number of antigens expressed by them. The antigens can be identified after they are specifically bound by surface receptors of vertebrate B and T immune cells (BCRs and TCRs, respectively). Because the vast repertoire of BCRs and TCRs cannot be encoded genetically, ancestors of jawed vertebrates adopted an elegant combinatorial solution [1]. The variable portions of the BCR and TCR genes are composed of separate V (variable), D (diversity), and J (joining) segments, which are represented by fewer than a few hundred copies each. In a B and T cell site-specific recombination reaction, commonly known as V(D)J recombination, one V, one D, and one J segment are joined together into a single exon encoding the variable antigen-binding region of the receptor. In addition to this combinatorial diversity, further diversity is generated by small insertions and deletions at junctions between the joined segments. In V(D)J recombination, DNA cleavage is catalyzed by two proteins encoded by the recombination-activating genes, approximately 1040-amino acid (aa) RAG1 and approximately 530-aa RAG2 [2,3]. The site specificity of the recombination is defined by the binding of RAG1/2 to RSSs flanking the V, D, and J segments [4]. All RSSs can be divided into two groups, referred to as RSS12 and RSS23, and consist of conserved heptamer and nonamer sequences separated by a variable spacer either 12 ± 1 (RSS12) or 23 ± 1 (RSS23) bp long [4–7].

During V(D)J recombination, RAG1/2 complex binds one RSS12 and one RSS23, bringing them into juxtaposition, and cuts the chromosome between the RSS heptamers and the corresponding V and D, D and J, or V and J coding segments [3,8]. A rule requiring that efficient V(D)J recombination occur between RSS12 and RSS23 is known as the “12/23” rule [1]. Even prior to the discovery of RAG1 and RAG2, it had been suggested that the first two RSSs were originally terminal inverted repeats (TIRs) of an ancient transposon whose accidental insertion into a gene ancestral to BCR and TCR, followed by gene duplications, triggered the emergence of the V(D)J machinery [4]. Later, this model was expanded by the suggestion that both RAG1 and RAG2 might have evolved from a transposase (TPase) that catalyzed transpositions of ancient transposons flanked by TIRs that were precursors of RSSs [9]. This model has received additional support through observations of similar biochemical reactions in transposition and V(D)J recombination [10,11]. Finally, it was demonstrated that RAG1/2 catalyzed transpositions of a DNA segment flanked by RSS12 and RSS23 in vitro [12,13] and in vivo in yeast [14]. In vertebrates, in vivo RAG-mediated transpositions are strongly suppressed, probably to minimize potential harm to genome function. So far, only one putative instance of such a transposition has been reported [15]. However, given the lack of significant structural similarities between RAGs and known TPases, the “RAG transposon” model [9,12,13,16] remained unproven. Here we demonstrate that the RAG1 core and RSSs were derived from a TPase and TIRs encoded by ancient DNA transposons from the *Transib* superfamily [17].

**Figure 1:**
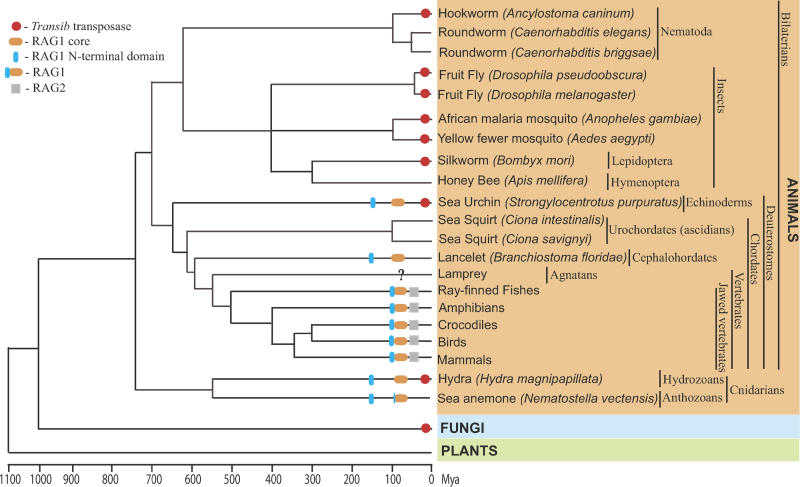
Schematic Presentation of *Transib* transposons, RAG1, RAG2, and RAG1-Like Proteins in Eukaryotes The basic timescale of the evolutionary tree is based on published literature [49–51]. Red circles mark species in which *Transib* TPases were found. Gray squares indicate RAG2; orange and blue ellipses show the RAG1 core and RAG1 N-terminal domain, respectively. Overall taxonomy, including common and Latin names, is reported on the right side of the figure. A question mark at the lamprey lineage indicates insufficient sequence data. A lack of any labels means that the *Transib* TPase and RAG1/2 are not present in the sequenced portions of the corresponding genomes. Among branches lacking *Transib* TPases, only lamprey and crocodile genomes are not extensively sequenced to date. In sea anemone, the RAG1 core–like protein is capped by the ring finger motif, which also forms the C-terminus in the RAG1 N-terminal domain. In fungi, the *Transib* TPase was detected in soybean rust only.

The *Transib* superfamily is one of ten superfamilies of DNA transposons detected so far in eukaryotes [17]. Like other DNA transposons, *Transib* transposons exist as autonomous and nonautonomous elements. The autonomous *Transib* transposons are 3–4 kb long and code for an approximately 700-aa TPase that is not similar to TPases from any other transposon superfamilies. Computational analysis of *Transib* elements, including their numerous insertions into copies of other transposons, demonstrated that *Transib* transposons are flanked by 5-bp target site duplications (TSDs), which also distinguishes this superfamily from all the others [17]. *Transib* transpositions are expected to be catalyzed by the binding of the TPase to TIRs of autonomous and nonautonomous transposons [17]. As discussed in this paper, in addition to the fruit fly *(Drosophila melanogaster)* and African malaria mosquito *(Anopheles gambiae)* genomes, in which *Transib* transposons were originally discovered, these genes are also present in diverse animals (Table S1), including other species of fruit fly (e.g., *Drosophila pseudoobscura, Drosophila willistoni*), yellow fever mosquito *(Anopheles aegypti),* silkworm *(Bombyx mori),* red flour beetle *(Tribolium castaneum),* dog hookworm *(Ancylostoma caninum),* freshwater flatworm *(Schmidtea mediterranea),* hydra *(Hydra magnipapillata),* sea urchin *(Strongylocentrotus purpuratus),* and soybean rust *(Phakopsora pachyrhizi).* Genomes of plants and vertebrates seem to be free of any recognizable *Transib* transposons (Figure 1).

## Results

### Detection of Similarity between *Transib* TPases and RAG1

Using protein sequences of seven known *Transib* TPases (Transib1 through Transib4 and Transib1_AG through Transib3_AG from D. melanogaster and *A. gambiae,* respectively) [17] as queries in a standard BLASTP search against all GenBank proteins, we found that the approximately 60-aa C-terminal portion of the Transib2_AG TPase was 35%−38% identical to the C-terminal portion of the RAG1 core (Figure S1). However, this similarity was only marginally significant (E = 0.07 where the E-value is an expected number of sequences matching by chance; Table 1). In another search against GenBank, using PSI-BLAST [18] (see Materials and Methods) with the Transib2_AG TPase as a query, we found that two unclassified proteins (GenBank gi 30923617 and 30923765; annotated as hypothetical proteins) and RAG1s constituted the only group of any GenBank proteins similar to the Transib2_AG TPase (Table 1). The statistical significance of similarity between the TPase and RAG1s was measured by E_i_ = 0.025, where E_i_ is the E-value threshold for the first inclusion of RAG1 sequences into the PSI-BLAST iterations [18] (Materials and Methods). The observed improvement in significance of the *Transib*/RAG1 similarity (from E = 0.07 in BLASTP to E_i_ = 0.025 in PSI-BLAST; Table 1) was due to the fact that both 151-aa and 123-aa hypothetical GenBank proteins were apparent remnants of *Transib* TPases (approximately 40% identity to the Transib2_AG TPase, E < 10^−10^ in BLASTP). The RAG1 proteins appeared to be more similar to the position-specific scoring matrix (PSSM) created by PSI-BLAST based on multiple alignment of the Transib2_AG TPase and two *Transib* TPase-like proteins, than to the solo Transib2_AG TPase in the BLASTP search.

**Table 1:**
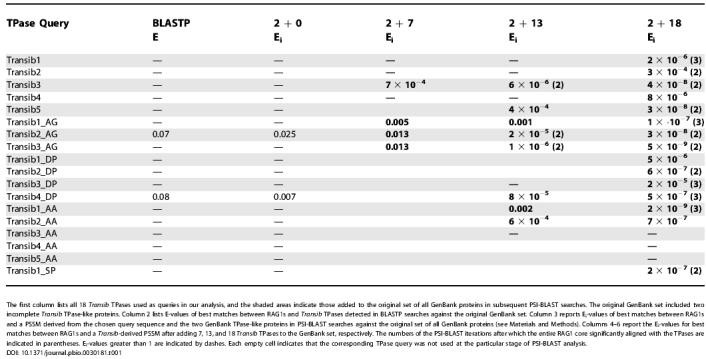
Significance of Similarities between the *Transib* TPases and RAG1 Core

The first column lists all 18 *Transib* TPases used as queries in our analysis, and the shaded areas indicate those added to the original set of all GenBank proteins in subsequent PSI-BLAST searches. The original GenBank set included two incomplete *Transib* TPase-like proteins. Column 2 lists E-values of best matches between RAG1s and *Transib* TPases detected in BLASTP searches against the original GenBank set. Column 3 reports E_i_-values of best matches between RAG1s and a PSSM derived from the chosen query sequence and the two GenBank TPase-like proteins in PSI-BLAST searches against the original set of all GenBank proteins (see Materials and Methods). Columns 4–6 report the E_i_-values for best matches between RAG1s and a *Transib*-derived PSSM after adding 7, 13, and 18 *Transib* TPases to the GenBank set, respectively. The numbers of the PSI-BLAST iterations after which the entire RAG1 core significantly aligned with the TPases are indicated in parentheses. E_i_-values greater than 1 are indicated by dashes. Each empty cell indicates that the corresponding TPase query was not used at the particular stage of PSI-BLAST analysis.

Given the latter observation, we decided to improve the quality of the PSSM constructed by PSI-BLAST for different *Transib* TPase sequences. To achieve that, we combined protein sequences of the seven known *Transib* TPases with the set of all GenBank proteins. As a result, E_i_-values for matches of RAG1s to a new PSSM based on alignment of nine *Transib* TPases (the two GenBank TPase-like proteins plus seven added TPases) noticeably dropped in comparison with the E_i_-values obtained for the PSSM constructed in the previous step based on alignment of the three TPases (Table 1).

To support the observation that E_i_-values of matches between RAG1s and the *Transib* TPase PSSM decrease as the number of TPase sequences used for construction of the PSSM increases, we identified six new *Transib* TPases (Transib5, Transib3_DP, Transib4_DP, Transib1_AA, Transib2_AA, Transib3_AA; Figure S2). During the next step of the PSI-BLAST analysis, the original GenBank set was combined with 13 *Transib* TPases. Again, E_i_-values of matches between RAG1s and the new PSSM derived from multiple alignment of 15 *Transib* TPases (the two GenBank proteins plus all our TPases) were much smaller (approximately 10^−6^–10^−3^; Table 1) than those obtained based on the PSSM constructed from the nine TPases at the preceding step (approximately 10^−3^–10^−2^). In the final step, we identified one more set of five new *Transib* TPases (Transib1_DP, Transib2_DP, Transib4_AA, Transib5_AA, and Transib1_SP). When all 18 TPases were combined with the original GenBank set, the E_i_ values of matches between RAG1s and the *Transib* PSSM dropped significantly further (10^−9^–10^−4^; Table 1). During the final revision of this manuscript, we identified an intermediate RAG1-like sequence in *Hydra magnipapillata,* called RAG1L_HM, which is significantly similar to both RAG1 and *Transib* TPase, as shown later. This direct result represents an independent validation of our analysis.

**Figure 2:**
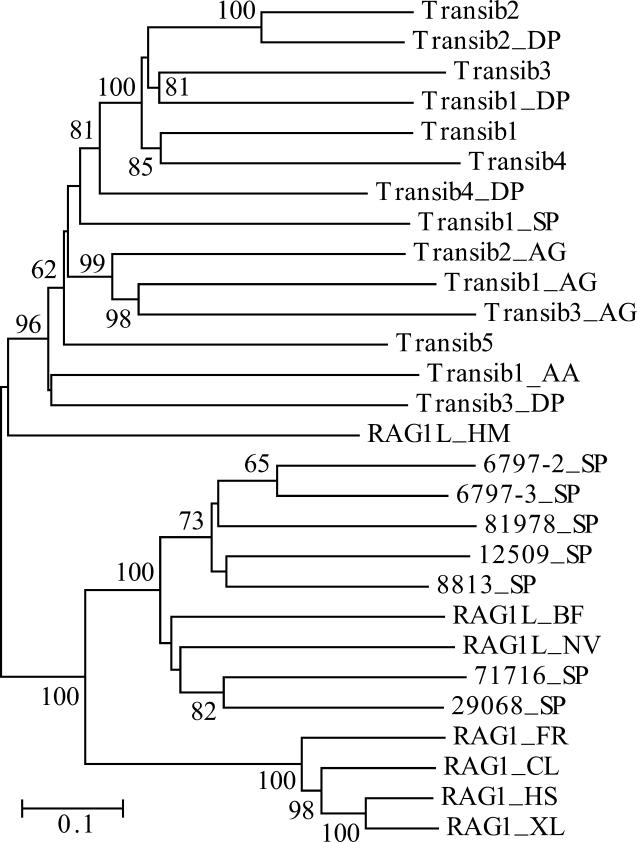
Diversity of the *Transib* TPases and RAG1 Core–Like Proteins in Animals The phylogenetic tree was obtained by using the neighbor-joining algorithm implemented in MEGA [44]. Evolutionary distance for each pair of protein sequences was measured as the proportion of aa sites at which the two sequences were different. Its scale is shown by the horizontal bar. Bootstrap values higher than 60% are reported at the corresponding nodes. Species abbreviations are as follows: AA, yellow fever mosquito; AG, African malaria mosquito; BF, lancelet; CL, bull shark; DP, D. pseudoobscura fruit fly; FR, fugu fish; HM, hydra; HS, human; NV, starlet sea anemone; SP, sea urchin; XL, frog. (Transib1 through Transib5 are from D. melanogaster fruit fly).

The PSI-BLAST PSSM of *Transib* TPases approximates conservation/variability of the *Transib* TPase consensus sequence. The more diverse the TPases used in determining the PSSM, the more accurate is the approximation; some of the insect *Transib* TPases are less than 30% identical to each other, as shown in Figure 2. The RAG1 E_i_ values decreased as the number of *Transib* TPases used for the PSSM construction increased due to the fact that RAG1 evolved from a *Transib* TPase. In all cases, the E values obtained after several rounds of iterations were less than 10^−20^ at the point of convergence. Nearly the entire sequences of several *Transib* TPases, excluding their 100–140-aa N-terminal domains, converged with an approximately 600-aa portion of RAG1 defined by positions approximately 360–1010 (Figure S3). This portion of RAG1 corresponds to the “RAG1 core,” hereafter numbered relative to human RAG1 (residues 387–1011), which along with RAG2 is known to be sufficient to perform V(D)J cleavage even after deletions of the 383-aa N-terminal and 32-aa C-terminal portions of RAG1 [19,20].

During studies reported here, we identified 11 additional new families of *Transib* transposons and TPases (see Figure S2) that are well preserved in the genomes of fruit flies (*Transib5* in *D. melanogaster;* and *Transib1_DP, Transib2_DP, Transib3_DP,* and *Transib4_DP* in *D. pseudoobscura)*, mosquitoes *(Transib1_AA, Transib2_AA, Transib3_AA, Transib4_AA,* and *Transib5_AA* from *A. aegypti)* and sea urchin *(Transib1_SP). Transib1_SP* is the first *Transib* transposon identified outside of insect genomes. A well-preserved 4132-bp *Transib1_SP* element (contig 7839, positions 376–4506) is flanked by a 5-bp CGGCG TSD, and it encodes a 676-aa TPase (two exons) that is most similar to the *Transib2* TPase (34% identity). Based on the currently available sequence data, we also reconstructed portions of TPases that were missed in previous studies [17] (Materials and Methods; see Figure S2). Using the Transib1_SP TPase as a query in TBLASTN searches against all GenBank sections (NR, HTGs, WGS, dbGSS, dbEST, dbSTS, and Trace Archives) we also found diverse *Transib* TPases in silkworm, red flour beetle, dog hookworm, freshwater flatworm, soybean rust, and hydra (Table S1). At the same time, recently sequenced genomes of honeybee, roundworms, fish, frog, mammals, sea squirts, plants, and fungi (except soybean rust) do not contain any detectable *Transib* transposons (see Figure 1). The observed patchy distribution could be caused by horizontal transfers and extinctions of *Transib* transposons in eukaryotic species.

### Common Structural Hallmarks of the *Transib* TPase and RAG1 Core

**Figure 3:**
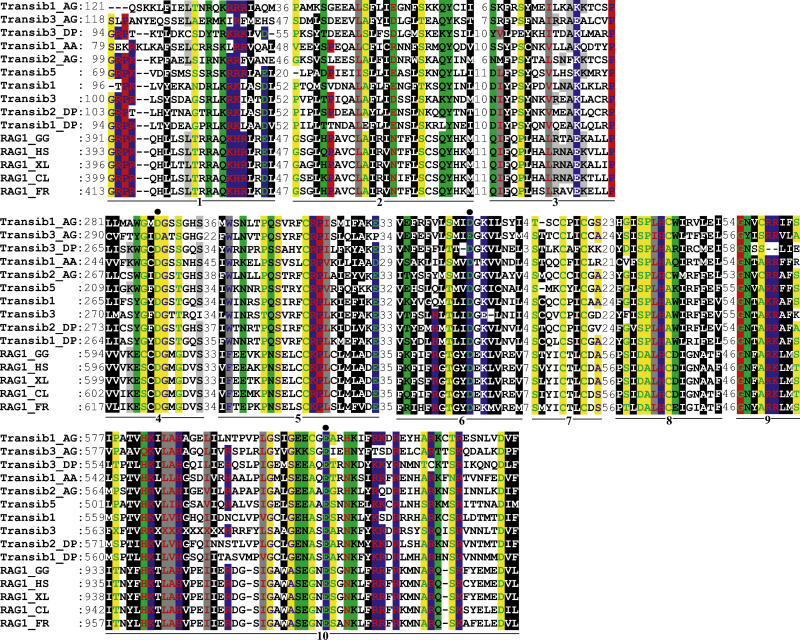
Multiple Alignment of Ten Conserved Motifs in the RAG1 Core Proteins and *Transib* TPases The motifs are underlined and numbered from 1 to 10. Starting positions of the motifs immediately follow the corresponding protein names. Distances between the motifs are indicated in numbers of aa residues. Black circles denote conserved residues that form the RAG1/*Transib* catalytic DDE triad. The RAG1 proteins are as follows: RAG1_XL (GenBank GI no. 2501723, Xenopus laevis, frog), RAG1_HS (4557841, *Homo sapiens,* human), RAG1_GG (131826, *Gallus gallus,* chicken), RAG1_CL (1470117, Carcharhinus leucas, bull shark), RAG1_FR (4426834, Fugu rubripes, fugu fish). Coloring scheme [43] reflects physiochemical properties of amino acids: black shading marks hydrophobic residues, blue indicates charged (white font), positively charged (red font), and negatively charged (green font); red indicates proline (blue font) and glycine (green font); gray indicates aliphatic (red font) and aromatic (blue font); green indicates polar (black font) and amphoteric (red font); and yellow indicates tiny (blue font) and small (green font). The species abbreviations for the Transib transposons are as follows: AA, yellow fever mosquito; AG, African malaria mosquito; DP, D. pseudoobscura fruit fly. *(Transib1* through *Transib5* are from the fruitfly *D. melanogaster)*.

**Figure 4:**
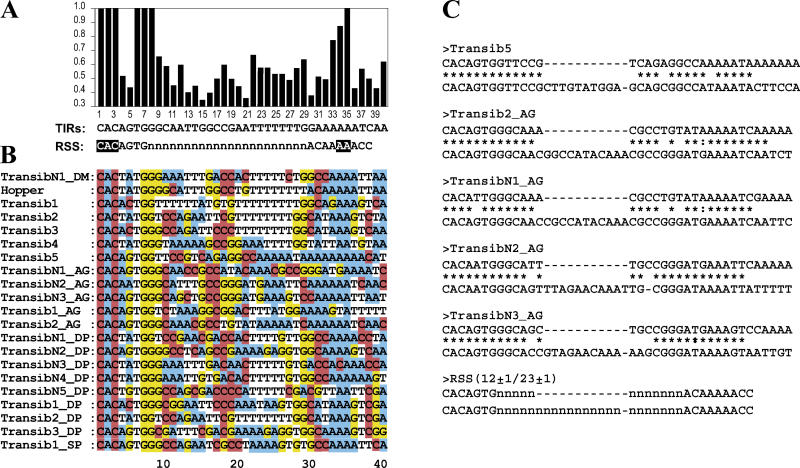
Structural Similarities between the *Transib* TIRs and V(D)J RSS Signals The species abbreviations are: AA, yellow fever mosquito; AG, African malaria mosquito; DM, D. melanogaster fruit fly DP, D. pseudoobscura fruit fly; SP, sea urchin. (*Transib1* through *Transib5* are from the fruit fly D. melanogaster). (A) Frequencies of the most frequent nucleotides at each position of the consensus sequence of the 5′ TIRs of transposons that belong to 20 families of *Transib* transposons identified in fruit flies and mosquitoes. The RSS23 consensus sequence is shown immediately under the TIRs consensus sequence. The most conserved nucleotides in the RSS23 heptamer and nonamer, which are necessary for efficient V(D)J recombination, are highlighted. The 23 ± 1 bp variable spacer is marked by Ns. (B) Non-gapped alignment of consensus sequences of 5′ TIRs from 21 families of *Transib* transposons*.* (C) The 12/23 rule follows from the basic structure of TIRs of the consensus sequences of transposons that belong to the *Transib5, Transib2_AG, TransibN1_AG, TransibN2_AG,* and *TransibN3_AG* families. The 5′ TIRs of these transposons are aligned with the corresponding 3′ TIRs. Structures of the 5′ and 3′ TIRs resemble RSS12 and RSS23, respectively.

All three core residues from the catalytic DDE triad in the RAG1 proteins (residues 603, 711, and 965) that are necessary for V(D)J recombination [21,22] are conserved in the *Transib* TPases (Figures 3 and Figure S3). This includes the distances between the second D and E residues, which are much longer in *Transib* transposons (206–214 aa) and RAG1 (253 aa) than in DDE TPases from other studied superfamilies (e.g., approximately 35-aa in *Mariner/Tc1* [23], 2-aa in *P* [23], approximately 35-aa in *Harbinger* [24], with *hAT* as an exception (325-aa, [25]). Moreover, each catalytic residue is a part of a motif that is conserved in the *Transib* TPases and RAG1 (motifs 4, 6, and 10 in Figures 3 and Figure S3). The RAG1 core is composed of the N-terminal region and the central and C-terminal domains ([26,27]. The N-terminal region includes the RSS nonamer-binding regions (residues 387–480), referred to as NBR [28,29]. The two terminal motifs of RAG1 NBR are conserved in the *Transib* TPases (Figure S3), which indicates that they may be important for their binding to the *Transib* TIRs during transposition (the RSS-like structure of TIRs is described below; Figure 4). The central domain of the RAG1 core (residues 531–763) includes two aspartic acid residues from the DDE triad and is also thought to be involved in binding to the RSS heptamer and RAG2 [30,31].

The C-terminal domain of RAG1 (residues 764–1011) is the portion of RAG1 that is most conserved between RAG1 and *Transib* TPases. In addition to the catalytic activity attributed to the last residue of the DDE triad, this domain has a strong nonspecific DNA-binding affinity because it binds to coding DNA upstream of the RSS heptamer, and is thought to be involved in RAG1 dimerization [26,27]. This domain is predicted to function analogously in *Transib* transposons. Several other motifs conserved in *Transib* TPases and RAG1 include aa residues that have been shown experimentally to be important for specific functions in V(D)J recombination (Figure S3). Based on this information, the function of these motifs in *Transib* TPases is expected to be similar to that in RAG1. Among the most conserved motifs, motif 5 (see Figures 3 and Figure S3) is of particular interest because its function is not known yet but is expected to play a role both V(D)J recombination and *Transib* transposition.

In conjunction with detailed studies of the *Transib* superfamily, we also analyzed the remaining nine known superfamilies of DNA transposons defined by diverse TPases (see Table 1 in [24]). Some of these TPases, including *Mariner, Harbinger*, *P*, and *hAT*, also contain the catalytic DDE triad [23]*.* However, based on PSI-BLAST searches, no significant similarities between these nine TPases and RAG1 protein were found (data not shown). Therefore, given that the only significant similarity of the RAG1 core was to the *Transib* TPase, the RAG1 core was re-confirmed as belonging to the *Transib* superfamily.

In addition to the statistically significant similarity between the approximately 600-aa RAG1 core and *Transib* TPases, there are two other lines of evidence suggesting evolution of the V(D)J machinery from *Transib* DNA transposons. They include the characteristic TSDs and structure of the TIRs discussed in the next two sections.

### Similar Length of TSDs and Target Site Composition in *Transib* and RAG1/2-Mediated Transpositions

It has been known that RAG1-mediated transposition in vitro, both intermolecular and intramolecular, is most frequently accompanied by 5-bp TSDs [12,13]. In one study [12], 35 of 38 (92%) TSDs generated during RAG-mediated intermolecular transposition were 5 bp long, and the remaining 8% were either 4 or 3 bp long. Also, 69% of 36 TSDs recovered during RAG-mediated intramolecular transpositions were 5 bp in length; of the remaining ones, 28% were 4 bp and 3% were 3 bp long. In another study [13], six of six TSDs detected in the intermolecular transposition were 5 bp long. Intramolecular transposition mediated by murine RAG1/2 proteins was also studied recently in vivo in yeast [14]. Again, 60% of TSDs recovered in 26 events were 5 bp long [14]. Given the predominance of 5-bp TSDs, it is striking that *Transib* transposons belong to the only superfamily of eukaryotic DNA transposons with 5-bp TSDs generated upon insertions into the genome [17,24]. To illustrate the characteristic 5-bp TSDs, we show copies of *Transib* transposons with intact 5′ and 3′ TIRs from diverse families of *Transib* transposons present in the *D. melanogaster, D. pseudoobscura, A. gambiae,* and S. purpuratus genomes (Figure S4). Moreover, some families show high target site specificity, e.g., *Transib-N1_AG* and *Transib-N2_AG* integrate preferentially at cCASTGg and cCAWTGc, respectively (TSDs are capitalized). RAG1/2-mediated transpositions also show significant target specificity, presumably reflecting the original specificity of the *Transib* TPase [12]. Indigenous properties of the *Transib* TPase, that were not related directly to RAG1 functions, including those responsible for the precise 5-bp length of TSDs, might have been altered during evolution of RAG1, leading to occasional 4-bp and 3-bp TSDs that are atypical for *Transib* transposons. Both RAG1/2-mediated and *Transib* transpositions show strong preference for GC-rich target sites [12–14,32], even though genomes hosting *Transib* transposons are AT-rich (Figure S4; Table 2).

### Structure of *Transib* TIRs

The structure and conservation patterns of the 38-bp termini of *Transib* transposons from 21 different families closely resemble those of RSSs, suggesting that the latter were derived from termini of ancient *Transib* transposons (Figures 4 and S4). The 38-bp consensus TIR of *Transib* transposons consists of a conserved 5′- CACAATG heptamer separated by a variable 23-bp spacer from an AAAAAAATC-3′ nonamer. This corresponds closely to the structure of RSSs, which are composed of the conserved heptamers 5′- CACAGTG separated by a variable 22-bp spacers from ACAAAAACC-like nonamers [1,5–7]. Only bases at positions 1 through 3 in the heptamer and at positions 5 and 6 in the nonamer are universally conserved in RSSs and absolutely essential for efficient V(D)J recombination [5–7]. The corresponding positions are perfectly conserved in all *Transib* transposons (Figure 4A and 4B; excluding the 85% conserved position 34 in the *Transib* consensus that corresponds to position 5 in the RSS nonamer). The probability of the observed match between the RSS and *Transib* termini to occur by chance is less than 10^−3^ (see Materials and Methods). Although most *Transib* families are represented by transposons flanked by TIRs similar to RSS23 (Figure 4A), several families include transposons with 5′ and 3′ termini similar to RSS12 and RSS23, respectively (Figure 4C). Therefore, even the 12/23 rule [1] can be derived directly from the sequence structure of known *Transib* transposons.

### RAG1 Core–Like Sequences in the Sea Urchin, Lancelet, Starlet Sea Anemone, and Hydra Genomes

**Figure 5:**
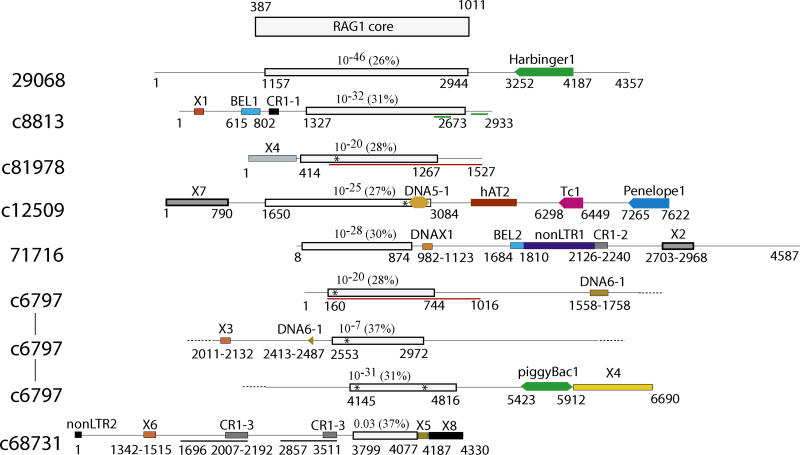
Schematic Structure of the Sea Urchin RAG1-Like Sequences Contig accession numbers are shown in the left column. Inverted complement contigs are marked by “c” followed by the contig number. In each contig, RAG1-like proteins (white rectangle) are schematically aligned with the human RAG1 core (top rectangle). Nucleotide positions of the RAG1-like sequences are shown beneath the white rectangles. Three pairs of recently duplicated sequences (nucleotide identity is higher than 95%) are underlined by red, green, and black lines, respectively. Transposable and repetitive elements detected in the flanking regions are marked by painted rectangles. Names of these elements are shown above the rectangles. Asterisks denote stop codons in the corresponding RAG1-like sequences. BLASTP E-values characterizing similarities between the sea urchin and RAG1 proteins are shown above the white rectangles. Multiple alignment of these protein sequences is reported in Figure S5.

Using RAG1 proteins as query sequences in a WU BLAST search against sea urchin contigs sequenced at Baylor College (see Materials and Methods), we identified eight proteins approximately 30% identical to portions of the RAG1 core and approximately 50% identical to each other (see Figures 2, 5, and S5). Only one protein is present in two copies, which are 94% identical to each other at the DNA level (contigs 81987 and 6797). Both copies appear to be encoded by pseudogenes damaged by a stop codon at the same position of each protein. Interestingly, the 6,690-bp contig 6797 harbours two additional defective pseudogenes coding for different RAG1 core–like proteins (Figure 5). We also identified a 597-aa protein sequence encoded by a single open reading frame (contig 29068, positions 1157–2944), which is 28% identical to nearly the entire RAG1 core (positions 461–1002 in the human RAG1, Figure S5). Extensive analysis of the flanks failed to show any hallmarks of putative transposons that might be associated with this RAG1-like protein, and we did not find any evidence indicating that other RAG1 core–like proteins are encoded by transposable elements (Figure 5).

**Table 2:**
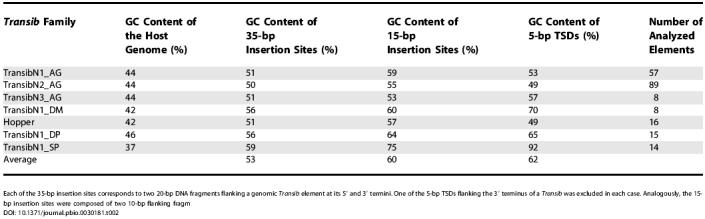
Preferential Insertion of *Transib* transposons into GC-Rich Sites

Each of the 35-bp insertion sites corresponds to two 20-bp DNA fragments flanking a genomic *Transib* element at its 5′ and 3′ termini. One of the 5-bp TSDs flanking the 3′ terminus of a *Transib* was excluded in each case. Analogously, the 15-bp insertion sites were composed of two 10-bp flanking fragm

Using FGENESH [33], we detected that the RAG1 core–like open reading frame (ORF) in the contig 29068 forms a terminal exon (positions 1154–2947) of an incomplete hypothetical gene composed of two exons (internal and terminal; see Figure S6). The 3′ terminal portion of the internal exon encodes a protein sequence that appears to be marginally similar to an approximately 50-aa fragment of the RAG1 core (positions 394–454 in human RAG1; Figure S5). The RAG1 core–like protein in whole genome shotgun (WGS) contig 12509 (Figure 5) also seems to be encoded by the last exon starting at position 1650 of a hypothetical RAG1-like gene. Although the two proteins are only 38% identical to each other, they share common features: (1) their N-terminal portions are missing and the RAG1-like sequences start at positions 17 or 18; (2) in both proteins the first aa residue overlaps with the acceptor splice site; and (3) their similarity to RAG1 starts at positions corresponding to position 470 of the human RAG1. Remarkably, the acceptor splice site positions in the sea urchin RAG1 core–like proteins closely correspond to those in RAG1 from teleosts (i.e., most of the living ray-finned or bony fish), in which RAG1 is split by an intron at position homologous to Gly_460_ in human RAG1 [34].

Using the same RAG1 query sequences in a TBLASTN search against WGS trace sequences from the lancelet *(Branchiostoma floridae)* genome recently sequenced at the Joint Genome Institute (see Materials and Methods), we found that the lancelet genome encodes protein sequences approximately 35% identical to the RAG1 core (Figure S5; RAG1L_BF; BLASTP E-value is equal to 10^−34^). Again, as in the case of the sea urchin sequences, the lancelet RAG1 core–like elements show no hallmarks of transposons (data not shown). However, unlike highly conserved RAG1 proteins, the RAG1 core–like proteins are remarkably diverse (see Figure 2).

During the second review of the manuscript of this article, we were kindly informed by Dr. Hervé Philippe of a RAG1 core–like sequence present the starlet sea anemone *(Nematostella vectensis).* After that, we screened all available Trace Archives (Materials and Methods) and detected additional RAG1-like proteins. In starlet sea anemone, several approximately 1000-bp WGS trace sequences were found (e.g., GenBank Trace Archive IDs 668021618, 558173651, 568641192, and 599572062), which encode protein, called RAG1L_NV, that is approximately 30% identical to the human RAG1 core (positions 284–802, TBLASTN, 10^−26^ < E < 10^−7^). We also found several approximately 1000-bp WGS trace sequences of Hydra magnipapillata (Trace Archive IDs 688654311, 647073738, 666995387, 687186526, 688683890, and 688948453), coding for protein sequences 26%−30% identical to the RAG1 core (positions 753−995, E-value is approximately equal to 10^−7^ in a BLASTX search against GenBank). Using these trace sequences, we partially assembled a hydra gene, called RAG1L_NM, which encodes the RAG1 core–like protein.

Remarkably, the hydra RAG1L_NM protein turned out to be significantly similar to the *Transib* TPase (26% identity; E-value is approximately equal to 10^−14^ in a BLASTX search against GenBank proteins combined with the *Transib* TPase sequences). Therefore, the hydra RAG1 core–like protein provides the first direct link between the RAG1 core and *Transib* TPase.

### N-Terminal–Like Domain of RAG1 in the Sea Urchin, Lancelet, Starlet Sea Anemone, and Hydra Genomes

**Figure 6:**
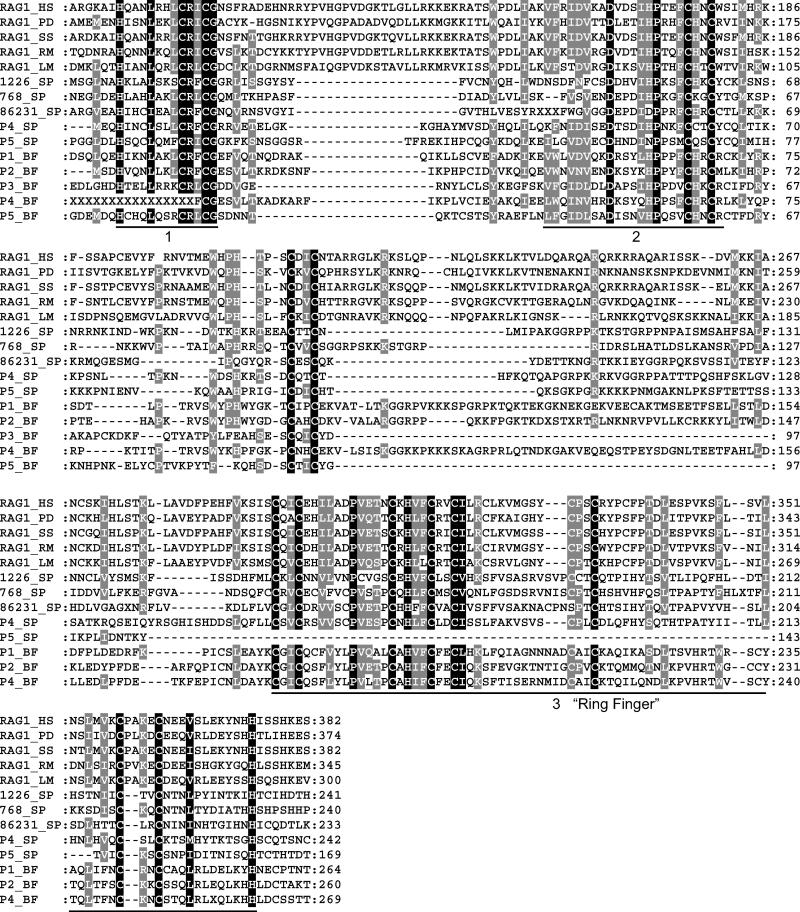
Multiple Alignment of the RAG1 N-Terminal Domain and Sea Urchin Protein Sequences RAG1_HS, RAG1_PD, RAG1_SS, RAG1_RM, and RAG1_LM mark the human (GenBank accession number NP_000439), lungfish (AAS75810), pig (BAC54968), stripe-sided rhabdornis or Rhabdornis mysticalis bird (AAQ76078), and latimeria (AAS75807) proteins, respectively. The sea urchin and lancelet proteins are marked by “_SP” and “_BF” following the identification numbers of the corresponding contigs. Protein sequences assembled from the sea urchin and lancelet WGS Trace Archives are denoted as P4-P5_SP and P1-P5_BF, respectively. Three conserved motifs are underlined and numbered. The third conserved motif is known as the ring finger. Distances from the protein N-termini are indicated by numbers.

A separate analysis of the assembled sea urchin sequences yielded seven sequences encoding three diverse proteins that were significantly similar to the 380-aa N-terminal domain of RAG1 (BLASTX, E < 10^−4^), excluding the 100-aa N-terminus (Figure 6). The first 305-aa protein is encoded by contig 1226, and its recently duplicated copies are on contigs 1219 and 1222 (approximately 95% identical to each other at the protein level.) The second, 195-aa protein (contig 83099) is the shortest. It is only approximately 26% identical to the first protein and more than 90% identical at the DNA level to its duplicate on contig 86231. We also found a third protein on contig 768 that contains unique motifs in its N-terminal regions that best match the homologous regions of RAG1. Furthermore, we found that unassembled WGS trace sequences encode two other proteins, P4_SP and P5_SP, similar to the N-terminal RAG1 domain (Figure 6).

By analyzing the lancelet WGS traces, we also found that the lancelet genome encodes five different proteins similar to the N-terminal domain of RAG1 (BLASTP E values in searches against all GenBank proteins were in a range of 10^−14^–10^−7^). DNA sequences coding for these proteins, P1_BF through P5_BF, were manually assembled from overlapping WGS sequences (data available upon request).

The proteins detected in the sea urchin and lancelet genome share a ring finger motif as well as two novel motifs matching the N-terminal RAG1 domain (Figure 6) and remotely resembling C-x2-C zinc finger motifs. The new conserved motifs are H-x3-L-x3-C-R-x-C-G and D-x3-I-h-P-x2-F-C-x2-C, and their function remains to be determined. It is thought that the ring finger motif of RAG1 functions as a zinc-binding domain, is involved in dimerization [30,35], and acts as an E3 ligase in the ubiquitylation [36]. It also likely that the N-terminal RAG1 and RAG1-like proteins share an additional conserved motif W-x-p-h-x(3–6)-C-x2-C that resides between conserved motif 2 and the ring finger (Figure 6).

None of the sea urchin and lancelet proteins align to the approximately 100-aa N-terminus of RAG1, which may indicate that this portion is missing from the genome or highly diverged and difficult to detect. It is also worth noting that this portion corresponds to a separate exon in some teleosts (see Discussion). The ring finger motif itself is also present in several sea urchin proteins unrelated to RAG1 but significantly similar to diverse proteins associated with immune and developmental systems as well as regulation of transcription. To test whether the reported sea urchin sequences represent a true RAG1-like match, we cut off the ring finger motif and repeated the BLASTP search against all GenBank proteins. Even without the finger, the remaining portions of the sea urchin sequences were significantly similar to the corresponding portions of RAG1. BLASTP E-values were 9×10^−9^, 7×10^−5^, and 10^−3^ for the P5_SP, P4_SP, and 768_SP sequences, respectively; because both the low-complexity filter and composition-based statistics were applied, the corresponding E-values were estimated very conservatively. BLASTP searches of the sea urchin sequences against all GenBank proteins, excluding RAG1, detected only the ring finger domain of the sea urchin sequences. E-values of these matches were much higher than the E-values of similarities to the RAG1 proteins (SP_768: 0.04 versus 7×10^−7^; SP_86231: 3·10^−4^ versus 7×10^−7^; SP_1226: 10^−4^ versus 2×10^−7^; P4_SP: 10 versus 2×10^−7^; P5_SP does not have ring finger and matches RAG1 only, E-value = 9×10^−7^).

Based on the same approach, our study found that the starlet sea anemone and hydra genomes also encode several families of the N-terminal RAG1 domain that appear to be separate from the RAG1 core–like proteins (data not shown). The only exception was the already mentioned sea anemone RAG1 core–like sequence. The approximately 90-aa N-terminus of the latter sequence is the ring finger (E < 10^−7^, multiple BLASTP matches against known ring fingers in GenBank).

## Discussion

The significant similarity between the *Transib* TPases and RAG1 core, the common structure of the *Transib* TIRs and RSSs, as well as the similar size of TSDs characterizing transpositions of *Transib* transposons and transpositions catalyzed by RAG1 and RAG2, directly support the 25-year-old hypothesis of a transposon-related origin of the V(D)J machinery. Previously, the “RAG transposon” hypothesis was open to challenge by alternative models of convergent evolution. Because there were no known TPases similar to RAG1, it could be argued that RAG1 independently developed some TPase-like properties, rather than deriving them from a TE-encoded TPase [24]. These arguments can now be put to rest.

As shown in this paper, the RAG1 core was derived from a *Transib* TPase, but given the low identity between the *Transib* TPase and the RAG1 core (14%–17%) it is not clear whether the ancestral transposon was a member of the group of canonical *Transib* transposons preserved in modern genomes of insects, hydra, and sea urchin (see Figure 1), or a member of an unknown group of *Transib* transposons that encoded a TPase that was more similar to RAG1 core than to the canonical TPase from the currently known *Transib* transposons. Furthermore, after its recruitment, the RAG1 core most likely went through a period of intensive transformations due to diversifying/positive selection, which further decreased its similarity to *Transib* TPase. Afterwards, the RAG1 genes continued to evolve at a slow and steady pace under stabilizing selection, as indicated by the observed conservation of the RAG1 core (79% identity between sharks and mammals).

Some of the intermediate stages of RAG1 evolution can be inferred from analysis of the sea urchin in which RAG1-like proteins were recently observed [37], and from analysis of the lancelet, starlet sea anemone, and hydra genomes. Based on the presence of stop codons disrupting some of the RAG1-like sequences, it has been suggested [37] that the sea urchin sequences represent remnants of transposable elements. Typically, TPase-coding autonomous DNA transposons are present in only a few complete copies per genome. At the same time, sequences homologous to their terminal portions, including specific TIRs, are usually abundant due to the proliferation of nonautonomous DNA transposons fueled by the TPase expressed by the corresponding low-copy autonomous elements. Therefore, even if only 30% of the sea urchin genome has been sequenced to date, it is expected that the regions flanking the TPase portions of potential autonomous elements should be similar to numerous nonautonomous elements. So far, we have found no evidence of such similarities. Detailed analysis of regions flanking the sea urchin RAG1-like DNA coding sequences revealed a variety of different transposable elements inserted in the proximity of the coding sequences (see Figure 5). Nevertheless, based on the orientations and relative positions of these transposons, none of them appears to be associated with the RAG1-like sequences (see Figure 5). We also could not identify the 5-bp TSDs and TIRs characteristic of the *Transib* superfamily. Still, given that only one third of the sea urchin genome is currently assembled as a set of contigs longer than several thousand nucleotides (the remaining portion is represented by short WGS sequences), we cannot rule out the possibility that the sea urchin RAG1-like proteins are remnants of an unknown branch of *Transib* transposons. Given that the genomes of lancelet, hydra, and starlet sea anemone are currently available only as unassembled WGS traces, the question whether the corresponding RAG1-like sequences are remnants of transposons or genes/pseudogenes must be left open.

The alternative possibility is that the sea urchin RAG1 core–like sequences represent diverse genes and pseudogenes that belong to a rapidly evolving multigene family. This opens the tantalizing possibility that the RAG1 core was recruited from a *Transib* TPase in a common ancestor of Bilaterians and Cnidarians, and subsequently lost in nematodes, insects, and sea squirts (see Figure 1). Furthermore, given that the sea urchin, lancelet, hydra, and starlet sea anemone genomes harbor several highly divergent N-terminal–like domains, separate from the RAG1 core–like sequences and known transposable elements, it is very likely that the N-terminal–like domains of RAG1 also form a multigene family that can be traced back to a common ancestor of Deuterostomes (see Figure 1). If so, then both N-terminal and core domains of RAG1 might have been derived from different genes present in a common ancestor of Deuterostomes. Alternatively, the N-terminal domain of RAG1 might have been derived from a separate, unknown transposon. The N-terminal domain of RAG1 has long been viewed as distinct from the core domain due to its lack of direct involvement in the V(D)J recombination reaction. In the sea urchin, lancelet, hydra, and starlet sea anemone genomes, the RAG1 core–like sequences and the N-terminal domain–like sequences do not appear to be linked to each other or to any other proteins. The only notable exception is the anemone RAG1 core–like protein sequence, which is capped by the 90-aa ring finger motif. Taken together with the fact that only the RAG1 core is significantly similar to *Transib* TPase, the data suggest that the vertebrate RAG1 represents a fusion of once separate proteins. This is consistent with the observation that in teleosts, (bony fish) the *RAG1* gene is divided into exons by either one or two introns. As a result, the RAG1 core is split into separate exons at the aa position that corresponds to position 460 in the human *RAG1*gene [29,34,38]. The core-like sequences encoded by the sea urchin WGS sequence contigs 29068 and 12509 correspond to either the second or third RAG1 exon in teleosts (depending on the number of introns), which is remarkably consistent with the fusion model. The same model predicts that the N-terminal domain of RAG1 could also be assembled from two separate domains based on the presence of the second intron in some teleosts, splitting the N-terminal domain into the 102-aa N-terminal subdomain and the rest [34]. As indicated above, this subdomain, corresponding to the first exon in the genes split by two introns, appears to be missing in the sea urchin, lancelet, hydra, and starlet sea anemone N-terminal–like proteins. It may be encoded by a separate exon that is difficult to detect given its short length and the high level of sequence divergence between these species and vertebrates, or it might have been added in vertebrates. Similarly, the RAG1 core–like protein in the sea urchin genome is shorter in its N-terminal part than the core domain in vertebrates and the corresponding *Transib* TPase. Again, it is unclear if this part is not present in sea urchins or simply undetectable due to its small size and the high sequence divergence.

It is currently believed that both RAG1 and RAG2 proteins were originally encoded by the same transposon recruited in a common ancestor of jawed vertebrates [3,12,13,16]. However, none of the *Transib* transposons identified so far encode any proteins other than the *Transib*/RAG TPase. Also, we could not find any RAG2-like sequences in the recently sequenced sea urchin, lancelet, hydra, and sea anemone genomes, which encode RAG1-like sequences. Autonomous DNA transposons from the *MuDR*, *Harbinger*, and *En/Spm* superfamilies are each known to encode a second regulatory protein [23,24], whereas some transposons from these superfamilies encode the TPase only. Therefore, it is in principle possible that an ancient vertebrate *Transib* that was a direct ancestor of the RAG1 core also encoded a second protein, the direct ancestor of RAG2. Nevertheless, the apparent lack of RAG2-like proteins in the sequenced portion of the sea urchin, lancelet, hydra, and sea anemone genomes, as well as in *Transib* transposons suggests that RAG2 was introduced in a separate event in jawless vertebrates. However, given the low 30% identity between the RAG1 and sea urchin/lancelet/sea squirt RAG1-like proteins, we cannot exclude the possibility that the ancestral RAG2 protein went through a period of strong diversification driven by positive selection, and it can no longer be identified by sequence comparisons but may still be present in invertebrates. In any case, the origin of the V(D)J recombination system in jawless vertebrates appears to be a culmination of earlier evolutionary processes rather than an isolated event associated with insertion of a single transposon. If so, detailed studies of individual components, including active *Transib* transposons and invertebrate proteins homologous to RAG1 elements can bring new breakthroughs in our understanding of evolutionary and mechanistic aspects of V(D)J recombination.

The observed sequence similarity between the RAG1 and *Transib* TPase protein can help to identify aa residues in the TPase that are crucial for transposition of *Transib* transposons. For instance, on the basis of the TPase comparison to RAG1 (see Figures S1 and S3), we were able to identify correct positions of the last two aa residues in the DDE catalytic triad (see Figure 2 in [17]), missed in our previous study due to insufficient data. Interestingly, only two cysteines of the zinc finger B (ZFB) C_2_H_2_ motif in RAG1 (residues 695–761) involved in its binding to RAG2 [30,31] are perfectly conserved in the *Transib* TPases (motif 7; see Figures 3 and Figure S3). The remaining portion of the ZFB motif was probably lost in TPases of insect *Transib* transposons, which do not encode RAG2-like proteins. Notably, two ZFB cysteines are part of the conserved SxxCxxC motif, and mutations of the serine from the same motif cause severe defects in RAG1 transpositions in vitro [32]. Therefore, the presence of serine in this motif is expected to be crucial to *Transib* transpositions.

After submission of our manuscript, additional biochemical evidence favoring evolution of V(D)J recombination from transposable elements was reported [25]. Analogously to V(D)J recombination, transposition of the fly *Hermes* transposon, which belongs to the *hAT* superfamily, is also characterized by a double-strand break via hairpin formation on flanking DNA and 3′ OH joining to the target DNA [25]. However, although the observed biochemical relationship between the *hAT* TPase and V(D)J recombination is a step forward in our understanding of transposition reaction, several arguments strongly suggest that V(D)J machinery evolved from a *Transib* rather than from *hAT* transposon. First, as we mentioned previously, there is no significant sequence identity between *hAT* TPases and RAG1, even if one employs a PSI-BLAST search with most relaxed parameters (i.e., E < 10, no filters, no composition-based statistics). Second, although RAG1/2-mediated transpositions are characterized by 5-bp (sometimes 4-bp) TSDs, all known *hAT* transposons are characterized by 8-bp TSDs. Third, unlike in the case of *Transib* transposons, TIRs of *hAT* transposons are different from RSS both in terms of DNA sequence similarities and their conservation patterns (Figure S7). Fourth, *hAT-* and RAG1/2-mediated transpositions differ dramatically in terms of the GC content of their target sites: Unlike *Transib* transposons and RAG1 transpositions occurring in GC-rich DNA, *hAT* transposons tend to be integrated into AT-rich regions (Table S2). All four arguments strongly favor evolution of V(D)J machinery from a *Transib* transposon. Most likely, the *Transib* transpositions are also characterized by hairpin intermediates formed by the ends of the donor DNA double-strand breaks, as observed during V(D)J recombination and *hAT* transposition.

## Materials and Methods

### 

#### DNA and protein sequences.

Assembled D. pseudoobscura sequences were downloaded from the Human Genome Sequencing Center at Baylor College of Medicine through the Web site at http://hgsc.bcm.tmc.edu/projects/drosophila/ on 2 March 2004. Preliminary A. aegypti sequence data were obtained from The Institute for Genomic Research through the Web site at http://www.tigr.org on 4 March 2004. Assembled D. melanogaster sequences were downloaded from the Berkeley Drosophila Genome Project at http://www.fruitfly.org/sequence/download.html on 17 February 2004. Partially assembled S. purpuratus contig sequences were downloaded on 12 August 2004 from the Baylor College of Medicine through the Web site at ftp://ftp.hgsc.bcm.tmc.edu/pub/data/Spurpuratus/blast/Spur20030922-genome. In addition to the assembled contigs, Baylor College of Medicine, Human Genome Sequencing Center (http://www.hgsc.bcm.tmc.edu) produced an approximately 8-Gb set of short unassembled WGS sequences, called “traces”, which cover nearly the entire sea urchin genome. We downloaded these sequences from the GenBank Trace Archive at the National Center for Biotechnology Information (NCBI; ftp://ftp.ncbi.nih.gov/pub/TraceDB/strongylocentrotus_purpuratus/) on 17 November 2004. Also, we downloaded an approximately 5-Gb set of unassembled traces that cover almost completely the 600-Mb genome of Florida lancelet (ftp://ftp.ncbi.nih.gov/pub/TraceDB/branchiostoma_floridae **/;** 3 December 2004). These sequences were produced and deposited in the GenBank Trace Archive by Department of Energy Joint Genomic Institute (http://www.jgi.doe.gov/). All other DNA and protein sequences were accessed from GenBank (NCBI) through the server at http://www.ncbi.nih.gov/Genbank/ and from Ensembl (EMBL-EBI and Sanger Institute) via the server at http://www.ensembl.org. Sequences of the *Transib1* through *Transib4* and *Transib1_AG* through *Transib3_AG* transposons [17] were obtained from the D. melanogaster (drorep.ref) and A. gambiae (angrep.ref) sections of Repbase Update [39] at Genetic Information Research Institute (http://www.girinst.org).

#### Sequence analysis.

Computer-assisted identification and reconstruction of the *Transib* transposons was done as described previously [17,40–42]. DNA sequence analysis including local sequence alignments, multiple alignments, and reconstruction of the *Transib* consensus sequences was done using software developed at Genetic Information Research Institute (available upon request) and WU-BLASTN 2.0 (http://blast.wustl.edu). To avoid background noise introduced by mutations, *Transib* relics, whose TPase-coding regions contained numerous stop codons and indels, were ignored unless several copies were available. (We included in the analysis incomplete relics of the *Transib2–5_AA* TPases represented by single DNA copies). Prediction of putative exons and introns encoded by the *Transib* consensus sequences was done with FGENESH [33] (at http://www.softberry.com). Multiple alignments of distantly related RAG1 and *Transib* TPase protein sequences were created by T-Coffee [40]. Shading and minor manual refinements of the aligned sequences were done using Genedoc [43]. Phylogenetic trees were produced by using MEGA3 [44]. Some of the sea urchin sequences encoding the RAG1 N-terminal domain were assembled from traces based on the Baylor BAC-Fisher server at http://www.hgsc.bcm.tmc.edu/BAC-Fisher **/** (the results of assembly were verified manually).

All GenBank proteins were downloaded from ftp://ftp.ncbi.nih.gov/blast/DB/fasta/nr (February 2004) and were combined into a single set with the identified *Transib* TPases. No *Transib* TPases had been deposited or annotated previously in GenBank, except for two short hypothetical proteins predicted automatically during annotation of the D. melanogaster genome: 151-aa gi:30923617 and 123-aa gi:30923765. These proteins are apparent fragments of *Transib* TPases encoded by relics of *Transib* transposons, including *Transib5_DM*.

A standalone 2001 version of PSI (Position-Specific Iterating)-BLAST [18,45] was used for detection of proteins that were significantly similar to TPases encoded by *Transib* and other superfamilies of DNA transposons. The PSI-BLAST program [18,45] is much more sensitive than a regular BLAST search due to the use of PSSM) PSI-BLAST first performs a standard BLASTP search of a protein query against a protein database and constructs a multiple alignment of matches exceeding a certain E-value threshold (called E_i_ value for the inclusion of sequences into PSI-BLAST iterations). From this alignment, a PSSM is constructed. The PSSM is a weight matrix indicating the relative occurrence of each of the 20 aa at each position in the alignment. This new PSSM is used as the score matrix for a new BLAST search in a second iteration. The process is repeated for a specific number of iterations or until convergence, when no additional proteins are added on successive iterations. The use of a PSSM in place of a fixed generic substitution matrix such as BLOSUM62 results in a much more sensitive BLAST search [18,45]. Important practical aspects of using PSI-BLAST were recently described [46].

To ensure that a conservation profile for the *Transib* TPases and *RAG1* proteins was not produced by a systematic error, we employed a procedure of “step-wise” PSI-BLAST iterations. In this procedure we studied dependence of E_i_ values on the number of the *Transib* TPases combined with the GenBank proteins. The following protocol describes the procedure: (1) Use a GenBank set combined with *N* number of *Transib* TPases (in our studies, *N* was equal to 7, 13, and 18), (2) run PSI-BLAST against GenBank combined with TPases using each TPase as a query or seed, (3) select only *Transib* TPase sequences with E-values less than 10^−5^ to define the PSSM, (4) take the best E-value (E_i_) obtained by PSI-BLAST for RAG1s when PSSM is constructed without RAG1, then (5) repeat these operations for different numbers (*N*) of TPases.

Significant convergence of RAG1 and *Transib* TPases was observed to be independent of the particular type of substitution matrix (the same result was observed for both BLOSUM62 and PAM70 matrixes). To avoid detection of false similarities caused by simple repeats and coiled coils, the PSI-BLAST search was performed using stringent conditions with the SEG [47] and COILS [48] filters masking all low-complexity regions and coiled coils, respectively; composition-based statistics [45] were also employed.

The probability P_1_ that the 5′ terminus of a transposon from a particular *Transib* family would match by chance an RSS at its most conserved positions (positions 1−3 in the RSS heptamer, and positions 5 and 6 in the RSS nonamer) was estimated based on the following formula: P_1_ = f_C_ × f_A_ × f_C_ × f_A_ × f_A_, where f_C_ (0.2) and f_A_ (0.3) are frequencies of C and A in a set of 38-bp 5′ termini of *Transib* transposons from 21 families (see Figure 4). The value of P_1_ is 0.001, indicating a significant similarity between *Transib* TIRs and RSS. Indeed, given that these five positions conserved in RSS are conserved in all TIRs from 21 families of *Transib* transposons, and the average identity between these 38-bp TIRs is only 49%, the chance of randomly matching these positions in TIRs from all 21 families is extremely small.

TBLASTN searches against the Trace Archive were performed by using the BLAST client (blastcl3 or netblast at ftp://ftp.ncbi.nlm.nih.gov/blast/executables/LATEST/, which accesses the NCBI BLAST search engine. Names of all available Trace Databases were taken from a list of databases at http://www.ncbi.nlm.nih.gov/blast/mmtrace.shtml.

## Supporting Information

Figure S1 Similarity between C-Terminal Portions of the *Transib2_AG* TPase and RAG1 Two examples extracted from the NCBI BLASTP output illustrate similarity between the approximately 60-aa C-terminal portions of the *Transib2*_*AG* TPase (which we used as a query in a BLASTP search against all GenBank proteins) and the RAG1 core.
(751 KB EPS).

Figure S2 Multiple Alignment of *Transib* TPases The catalytic DDE triad is marked by black rectangles. Amino acids are shaded on the basis of their physiochemical properties according to the color scheme implemented in Genedoc [43]: Black shading marks hydrophobic residues, blue indicates charged (white font), positively charged (red font), and negatively charged (green font); red indicates proline (blue font) and glycine (green font); gray indicates aliphatic (red font) and aromatic (blue font); green indicates polar (black font) and amphoteric (red font); yellow indicates tiny (blue font) and small (green font). The species abbreviations are as follows: SP, sea urchin; DP, D. pseudoobscura fruit fly; AG, African malaria mosquito; AA, yellow fever mosquito. *Transib1* through *Transib5* are from the D. melanogaster fruit fly genome.
(3 MB EPS).

Figure S3 Multiple Alignment of the RAG1 Core and *Transib* TPase Proteins The shading scheme is the same as in Figure S2. The catalytic DDE triad is marked by black rectangles. RAG1 aa whose replacements resulted in previously detected defects of V(D)J recombination [31] are marked by color rectangles indicated below the alignment blocks; red indicates DNA binding defect; green indicates nicking defect; cyan indicates hairpin defect; blue indicates joining mutants; yellow indicates catalytic mutants; gray indicates joining/transposition. Presence and absence of corresponding residues in the *Transib* TPases are indicated by + and −, respectively. Conserved motifs are marked by lines numbered from 1 to 10. The species abbreviations are as follows: DP, D. pseudoobscura fruit fly; AG, African malaria mosquito; AA, yellow fever mosquito; GG, chicken; HS, human; XL, frog; CL, bull shark; FR, fugu fish.
(3 MB EPS).

Figure S4 TSDs in Transposons from Different *Transib* Families For each family, DNA copies of transposons are aligned to the corresponding consensus sequence. The consensus sequence is shown in the top line. Dots indicate nucleotide identity with the consensus sequence; hyphens represent alignment gaps. Internal portions of transposons are not shown and are marked by xxx. TSDs are highlighted. Coordinates of the reported elements are shown in the first two columns (sequence name, beginning to end).
(A) *TransibN1_AG* family from mosquito.
(B) *TransibN2_AG* family from mosquito.
(C) *TransibN3_AG* family from mosquito.
(D) *TransibN1_DP* family from fruit fly.
(E) *Hopper* family from fruit fly.
(F) *TransibN1_DM* family from fruit fly.
(G) *TransibN1_SP* family from sea urchin.
(179 KB PDF).

Figure S5 Multiple Alignment of the RAG1 Core and RAG1 Core–Like Proteins Encoded by the Sea Urchin and Lancelet Genomes The shading scheme is the same as in Figure S2 and S3. The species abbreviations are as follows: SP, sea urchin; BF, lancelet; HS, human; CL, bull shark; GG, chicken; XL, frog; FR, fugu fish. The lancelet RAG1L_BF protein is encoded by several overlapping WGS trace sequences (for example, GenBank Trace Archive identification numbers 543943730, 538583629).
(2.8 MB EPS).

Figure S6 RAG1-Like Protein SP_29068 in the Sea Urchin Contig 29068 (A) Exon/intron structure of the *SP_29068* gene is reported based on the FGENESH prediction.
(B) Alignment of the predicted protein and human RAG1 (29% identity, E = 10^−43^. The intron in *SP_29068* is inserted between residues shaded in green and red. Gly_460_ that harbors the intron in the teleost *RAG1* is shaded in black.
(1.5 MB EPS).

Figure S7 Structure of *hAT* 5′ Termini Non-gapped alignment of consensus sequences of 5′ termini of transposons from 22 different families is shown beneath the RSS23 consensus sequence, composed of the RSS heptamer and nonamer. The most conserved nucleotides in the heptamer and nonamer, which are necessary for efficient V(D)J recombination, are highlighted. Among the necessary RSS nucleotides, only one, marked by a + corresponds to a nucleotide that is 100% conserved in *hAT* transposons. The critical third nucleotide of the *hAT* 5′ termini is always G, as opposed to C in the RSS heptamer. It is also clear from the alignment that the *hAT* termini do not have any second conserved block, which is expected to be preserved if RSSs have evolved from *hAT* termini. *Hobo* (GenBank number X04705), *Homer* (AF110403), *Hermes* (L34807), *Ac9* (K01904), *Tam3_AM* (X55078), *TAG1* (L12220), *Pegasus* (U47019) are active *hAT* transposons from fruit fly, Queensland fruit fly, house fly, maize, snapdragon, thale-cress, and African malaria mosquito, respectively. *HOPPER_BD* is from oriental fruit fly (GenBank AF486809). The consensus sequences of *hAT-1N_DP* and *hAT-1N_DP* (nonautonomous transposons from fruit fly, D. pseudoobscura); *HAT1N_DR, hAT-2n1_DR,* and *hAT-N19_DR* (nonautonomous transposons from zebrafish); *CHARLIE1A* and *CHESHIRE* (human); *hAT-N1_SP* (sea urchin); *ATHAT1, ATHAT7,* and *ATHAT10* (thale-cress); *PegasusA, HATN4_AG,* and *hAT-2N_AG* (African malaria mosquito) were reported in Repbase Update.
(775 KB EPS).

Table S1 *Transib* TPase in Eukaryotes Columns 1 and 2 list common and Latin names of species whose genomes contain *Transib* TPase sequences. Column 3 shows GenBank sections collecting corresponding sequences: "NR", "WGS", "EST", and "HTGS" are names of GenBank sections; "tr" stands for “Trace Archives.” Column 4 shows a range of E-values of matches between the sea urchin *Transib* TPase (Transib1_SP) and TPases encoded by the listed species that were detected in TBLASTN searches against corresponding sections of GenBank. Matches to the *Transib* TPase observed for *Oryza sativa indica* (seven sequences from Trace Archives, 10^−48^ < E < 10^−13^) were discarded as a likely sequencing contamination, based on the fact that these sequences were over 80% identical to Hydra magnipapillata traces (the hydra Trace Archive dataset contains over 100 sequences matching the TPase, and hydra *Transib* TPase sequences are also present in the dbEST section of GenBank). Analogously, matches to the *Transib* TPase detected in the AC011430 HTGs and AADC01054609 WGS GenBank sequences, which were annotated as portions of the human genome, were discarded as products of contamination (these sequences contain 100% identical copies of the non-long terminal repeat (LTR) retrotransposon *G2_DM* [17] from D. melanogaster).
(27 KB DOC).

Table S2 GC Content of Target Sites for *hAT* Transposons The table shows that *hAT* transposons are inserted preferentially into GC-rich sites. Each of the 35-bp insertion sites corresponds to two 14-bp and 13-bp DNA fragments flanking a genomic *hAT* element at its 5′ and 3′ termini; one of the 8-bp TSDs (flanking the 3′ terminus of a transposon) was excluded in each case. Analogously, the 15-bp insertion sites were composed of two 4-bp and 3-bp flanking fragments. (1) GenBank accession number U47019; (2) Repbase Update, the angrep.ref section; (3) GenBank X04705; (4) Repbase Update, the drorep.ref section; (5) Repbase Update, spurep.ref; (6)Repbase Updates, the zebrep.ref section. Copies of *Pegasus, HATN4_AG, and HAT2N_AG* were identified in the mosquito A. gambiae genome; *Hobo* and *hAT-1N_DP* in the D. melanogaster and D. pseudoobscura fruit fly genomes, respectively; *HAT-1N_SP* in the sea urchin genome; and *HAT1N_DR, HAT-2N1_DR,* and *HAT-N19_DR* in the zebrafish genome.
(27 KB DOC).

### Accession Numbers

The sea urchin *Transib1_SP* transposon, *RAG1L_HM, RAG1L_BF, RAG1L_NV, 81978_SP, 12509_SP, 6797–1_SP, 6797–2_SP, 6797–3_SP, 8813_SP, 71716_SP, and 29068_SP* genes/pseudogenes have been deposited on our website (http://girinst.org/server/publ/PLOS.2005) and in the Third Party Annotation (TPA) database of GenBank (http://www.ncbi.nih.gov/Genbank/TPA.html; accession numbers are pending. The *Transib1, Transib2, Transib3, Transib4, Transib1_AG, Transib2_AG, Transib3_AG, Transib1_DP, Transib2_DP, Transib3_DP, Transib4_DP, Transib1_AA, Transib2_AA, Transib3_AA, Transib4_AA, Transib5_AA, Transib1_SP, TransibN1_SP, TransibN1_AG, TransibN2_AG, TransibN3_AG, TransibN1_DM, TransibN1_DP, TransibN2_DP, TransibN3_DP, TransibN4_DP,* and *TransibN5_DP* transposons are deposited in the drorep (D. melanogaster), angrep (A. gambiae), spurep (S. purpuratus), and invrep (invertebrates) sections of Repbase Update (http://www.girinst.org/Repbase_Update.html).

## Abbreviations

aa: amino acid
BCR: B cell receptor
E_i_-value: E-value threshold for the first inclusion of matching sequences into the PSI-BLAST iterations
NCBI: National Center for Biotechnology Information
PSI-BLAST: position-specific iterated BLAST
PSSM: position-specific score matrix
RSS: recombination signal sequence
TCR: T cell receptor
TIR: terminal inverted repeat
TPase: transposase
TSD: target site duplication
WGS: whole genome shotgun
ZFB: zinc finger B
