# Supplementary material for: RAG1 Core and V(D)J Recombination Signal Sequences Were Derived from Transib Transposons
Source: PLoS Biol. 2005 May 24;3(6):e181. doi: 10.1371/journal.pbio.0030181 (PMC1131882; doi:10.1371/journal.pbio.0030181)
Supplement: Supplementary file 1 [file pbio.0030181.sg004.pdf]

Figure S4a.

```
TransibN1_AG          :                     CACATTGGGCAAACGCCTGTATAAAAATCGAAAAxxxGAATTGATTTTCATCCCGGCGTTTGTATGGCCGTTGCCCACTGTG
2L  5119823-5118806   : CCGGCCGAGACCGGCCAATG..................................xxx......G........................G.............CAATGGTTGCAATGGGGGCA
2L  8712511-8711509   : GGACTGATTACCTATCAGTG...G....A.........................xxxA.................A............T.............CAGTTGTATAATTAGGCTTT
2L 20018136-20017277   : AGAACCAGTTGGAACCAGTG..................................xxx..........................................C..CATTGGAGGCGTCCGGAGCC
2L 20114414-20115455   : TTGACGTGGCAAAGCCAATG..................................xxx.............................................CAATGGTGAGACTGCAAGGC
2L 23799431-23798415   : GGTTACGAAACACGCCACTG...................A..............xxx.............................................CACTGGTGTTTCGAGTAGCA
2L 26104831-26103068   : AATTTACTCTTTCCCCACTG..................................xxx...............................G.............CACTGGCGGGGCTAAGGATA
2L 26230738-26229720   : GCTCATGAATTCCACCAGTG..................................xxx.............................................CAGTGTGACGTCGTCCCTGG
2L 27942244-27943258   : ACACGAGACTGCGCCCACTG..................................xxx...............................G.............CACTGGAACTGTCTACTCTC
2L 29228399-29227384   : CACGCATCCTCTCGCCATTG..................................xxx...............................G.............CATTGGTAAAAGTAGCAATC
2L 46313828-46312914   : TGTGCGCTGAACGGCCAGTG..................................xxx.............................................CAGTGAAAGGTCGCTCCGGT
2R  8144738-8145756   : GAAGCGAAGCCACACCAGTG..................................xxx.............................................CAGTGTCACCTTCTTGCACA
2R 11369580-11368562   : GAGCAGCAGCACCACCATTG..................................xxx.............................................CATTGGGGCGATATGATACG
2R 22247416-22246400   : AAGCACGGCGCTCACCCGTG..................................xxx.............................................CCGTGGAGAGAAAGCAAGGC
2R 22299245-22300260   : TCAGGGATGAGACACCAGTG..................................xxx.............................................CAGTGGAGGTGTTAAGTCAG
2R 27175422-27174152   : TACGCCCCGACCGTACACAG......................G...........xxx-----...............A..........G.............CACAGGGACCATTGAACTCC
2R 30585530-30586513   : GAAACGCACCGGCACCACTG..................................xxx...............................G.............CACTGGAAGGAAAGCCACCA
2R 39256338-39255331   : CGGACGCTTTTCGGCCAGTG..................................xxx.............................................CAGTGGTGTGTCCTATCCGT
2R 42675394-42674267   : TTAAACTATTGGAACCTGTG..................................xxx.............................................CTGTGGAGGTACCGTGTGGA
2R 43075924-43076941   : ATTTTTTTCTTCTTCCATTG..................................xxx...............................G.............CATTGGCCCTTCCGAAGAAT
2R 45171313-45172328   : ATTGCAAATGGCAGACACTG..................................xxx.C.............................G.............CACTGGGGCCTTAATTTTTC
2R 52938105-52937140   : TGGAGTGTGTCCTGACAATG..................................xxx........................A....................CAATGGAGAGAGGAAGATAG
2R 58350365-58349349   : GCTCACTCAGCTCACCATTG..................................xxx.............................................CATTGATGTAAGGAGGAAGC
2R 59515839-59516855   : ACATGACTAGCCCACCACTG..................................xxx...............................G.............CACTGCCCAGAGAGTATACT
2R 60219464-60220478   : CAGGGTACTCTTTACCTATG..................T...............xxx...............................G...T.........CTATGGTTCCGCAAAAATCT
2R 60905378-60904363   : CAACGCCAGCAGCACCACAG..................................xxx.................T...........................CACAGGCGAGACAGCTTTCC
2R 62298466-62296973   : GAGCCATACTTTTATCAGTG......A...........................xxx........................T....................CAGTGGTCGTATAGTTTATT
3L  6940556-6941406   : AACAGTTGTTGCGACCAGTG..................................xxx.............................................CAGTGTTGCGAACGGTGATA
3L 13551031-13552046   : TGTCGATTTTACCCCCATTG..................................xxx...............................G.............CATTGGCCACACAAGTTTTA
3L 16304955-16305971   : TCCACTGTAACGGACCACTG......................G...........xxx...............................G.............CACTGACGAGGAGGAACAGG
3L 20046623-20047640   : CATAGCTTTTGCTCCCAATG..................................xxx.............................................CAATGGTAGATCCATTTAAC
3L 20346726-20347741   : AAACATTTGTTGTACCACTG..................................xxx...............................G.............CACTGTACACAGAAGAATTC
3L 24406891-24405877   : TTGAGCTATTGCAATCATTG..................................xxx.............................................CATTGGAGCTAACCGGAACC
3L 39688132-39689148   : GGGCTCTCGTCTCCCCAATG..................................xxx.............................................CAATGTCGACAGTTTTTCAT
3R 28454268-28453252   : TGGTAGTTGTGCTACCACTG..................................xxx...............................G.............CACTGGTCAATGTCCAGGGG
3R 28657488-28658505   : AGGGAGCAGTGCCCGCACTG..................................xxx.............................................CACTGGGGAGAGGCATTGAA
3R 29529360-29528379   : GGCCGGATCTTCCACCACTG..................................xxx...............................G.............CACTGGTCCCTTCCTTCATT
3R 37506679-37505664   : GGGCAGCTTCCACTCCACTG..................................xxx.............................................CACTGTGCAGTCATAGTCAT
3R 41072498-41073486   : ATTAGCCGGTGAAGACACTG..................................xxx......G........................G.............CACTGGGAAGGATAAGGTTA
3R 51768770-51767266   : ATCTTTAGACGAAGTCATTG....................C.............xxx.............................A.....A.........CATTGGTGCTATCAAAAAAC
U   7556694-7555780   : TGCACGCAGGCCTTTCAATG..........................C.......xxx.............................................CAATGGCGTGCACTGACGAA
U  11176774-1117791   : GACGCGAGCCGCTACCATCG..................................xxx.............................................CATCGTCCACGCCCGAGAGA
U  14010308-14011323   : ATCGGTTACTGCCACCAACG......................G...........xxx...................T...........G.............CAACGGCGAGACGCTGTTGA
U  14253260-14254261   : CCCCGCTGCACGTACCAGTG..................................xxx.............................................CAGTGATGTGGCTGTGCCCG
U  23855743-23856761   : GGTAATGCGTCCCCCCAGTG..................................xxx..........C..................................CAGTGCCACCGTAATTAGGA
U  28858008-28856995   : TTTAATTAGCTGGACCACTG..................................xxx...............................G.............CACTGCCCTCGCGATCAGTG
U  30138472-30136500   : CTGAAAATCACCCACCAGTG...........C......................xxx...............-T............................CAGTGGTACGTGGAAAACTT
```

Legend to Supplementary Figure S4a. Alignment of mosquito *TransibN1_AG* elements to the *TransibN1_AG* consensus sequence. The consensus sequence is shown in the top line. Dots indicate nucleotide identity with the consensus sequence; hyphens represent alignment gaps. The internal portion of *TransibN1_AG* is not shown and is marked by xxx. Target site duplications are highlighted. Coordinates of the reported elements are shown in the first two columns (sequence name, beginning-end).

Figure S4b.

```
TransibN2_AG        :                     CACAATGGGCATTTGCCGGGATGAAATTCAAAAAxxxAAAAATAATTTTATCCCGCAATTTGTTCTAAACTGCCCATTGTG
2L  1229206-1230963 : AGTCAGTAACTATCTTAATG..................................xxx............................................TAATGGGGAGCCCATGAACA
2L  2089616-2091530 : ATATTTTTGCCGGTTCATTG..................................xxx..................A........A................CATTGGTATGGAGCGCTCTC
2L  2170283-2172340 : AAATTTTTACCCTTCCATTG..................................xxx............................................CATTGCTGATAGTATGGAAA
2L  2252107-2253892 : TGTAAGCCGGTATCACAATG..................................xxx............................................CAATGATGCGGTTCGATTGG
2L  4182055-4184049 : ATGGAGGAGCCGCTATAATG..................................xxx................T...........................TAATGACGAGCTATACGAGA
2L  4573850-4571975 : TCTCGACATCTGACTCAGTG..................................xxx..................AT........................CAAGTGGTTCGCTTACGTTT
2L  7014493-7012680 : TTCCACTTTTCATACCAGTT..................................xxx..................A.........................CAGTTGTATACAAAAGAGCC
2L 10734918-10737530: CATTTTACAGCACACCATTC..................................xxx............................................CATTCATCTCTCAGCAAAAA
2L 17020534-17018712: CAATAACATGCCCGCCATGG............................A.....xxx..................................T.........CATGGGTTCAAGCGTCAAAC
2L 20182158-20183951: TCGAGAATGACTTGCCATTG..................................xxx............................................CATTGTGGCAACCAAAACGC
2L 21232263-21234033: CCCAGCCAAGCCCGCCATTG..................................xxx.....AT.....................................CATTGAAGGAAAACCAACAA
2L 23139669-23140800: CCGTGAGATAGCCACCAATG..................................xxx..................A.........................CAATGAGCTGGTGGAGCCCA
2L 24782750-24784569: TTCGTCAGAGTTGATCAATG.................C................xxx............................................CATTGGTCGTAATTTTATAA
2L 32394929-32396712: ATACATATTTGCCAGCCTTG..................................xxx............................................CCTTGGTGTGATGGGGTGGG
2L 42315451-42313664: TAGAACGAACCCCCCCAGTG..................................xxxG...........................................CAGTGGAGCGATTTGAACAC
2R  2723033-2721042 : GCAGGAAAGCCCAACCGGTG..................................xxx............................................CGGTGGGACGGTTGGGCAGC
2R  5088053-5086059 : CCCCCATATCCTCTTCACAG..................................xxx............................................CACAGACACAGTCGTACTAC
2R  7767923-7766035 : ACCGGACGAGGACACCAGTG..................................xxx..................A.........................CAGTGGCCAGCGACCGGGTG
2R  9756593-9758384 : CTTTCGATGTGCCGGCACTG..................................xxx............................................CACTGGTGGCAGCAAACAGT
2R 12786847-12788637: TCGCATGTGCCCTGCGAATG..................................xxx............................................GAATGGTGCAAATTGTGTTC
2R 18717091-18715307: GCATACCACCCACCCCAGTG..................................xxx............................................CAGTGGTGGCGTAGTGCTTC
2R 19142821-19140572: CGTTGCCCTTCATCCGATTG..................................xxx..................A.........................GATTGGCAGCAATCCCCCCA
2R 25221892-25220094: CCTAAGCTCCCACCCGAATG..................................xxx............................................GAATGGTCTAAATATGGGAG
2R 29487916-29486129: CACTAATACTCTCGTCATTG..................................xxxG...........................................CATTGGCGTCATCGTTTGGA
2R 29903707-29901922: AGCAAAACTCCCCACCATTC..................................xxx............................................CATTCGCATAATTTATCACA
2R 40734221-40732243: GGCTACATTTGATCTCATTG..................................xxx............................................CATTGGCAAGGTCTCGCAAC
2R 41484089-41482326: TTCTTTCACCCCCACCACGT..................................xxx............................................CACGTGTGAATGGAAAGGAA
2R 44838408-44836585: ACAACATAGCCGTGCCAATG..................................xxx............................................CAATGAAAAAAAAACGCAAA
3L 27660824-27659035: TGTTGATTACTTCTACCATG..................................xxx............................................CCATGGTGAGCTCTAGTTTA
3L 29505347-29507134: ACGAAAAAGGTGCACCATTG..................................xxxG...........................................CATTGGACCACAATTTCATT
3L 34962407-34964402: AAGAAGGAACCAATCCAGTG..................................xxx............................................CAGTGTCCCACTGCTATGAG
3L 40833349-40831590: ACACACAGGGCCGATCAATG..................................xxx..................A.........................CAATGGGCCAGGTTGCATAG
3R   662089-660298  : TTTAACACTCTACACCAGTG..................................xxx............................................CAGTGATTTCACCATACACA
3R  7439751-7441730 : AAAACTTACTCCTCCCATCG..................................xxx............................................CATCGGCGTGGTTTGATTTA
3R 21327992-21326192: AGTGTGTTTGTGTGACACTG..................................xxx............................................CACTGTTAGGCTGGCGGGAT
3R 25880487-25882264: GAGCGAATCGGGCGACACTG..................................xxx.....A......................................CACTGGTTGGCTTCGGAGCG
X  11068447-11070272: CCAATACAATCATTACATTG..................................xxx.......T..........A.........................CATTGGTGAGTTTCAACCGT
X  12420031-12422325: TCCGTGCGCCTCGCCAATGC................A................xxxG.................A.........C...............CAATGTGCCGGTCCTGTGCA
X  15942596-15940943: TTATTGAAATCGCCACATAG.G..........................A.....xxx..................A.........................CATTGGTGTACAGCGTGTGA
```

Legend to Supplementary Figure S4b. Alignment of mosquito *TransibN2_AG* elements to the *TransibN2_AG* consensus sequence. The consensus sequence is shown in the top line. Dots indicate nucleotide identity with the consensus sequence; hyphens represent alignment gaps. The internal portion of *TransibN2_AG* is not shown and is marked by xxx. Target site duplications are highlighted. Coordinates of the reported elements are shown in the first two columns (sequence ID, beginning-end).

Figure S4c.

```
TransibN3_AG         :                    CACAGTG-GGCAGCTGCCGGGATGAAAGTCCAAAAxxxACTTTTATCCCGC---TTTTTGTTCTAC-GGTGCCCACTGTG
2L  6093702-6094171 : AGGTGATGCTCAAACAGTTG.......-..................-.C....C.xxx............G---............-....T........AGTTGGTGTGATCAATACGT
2L 38028097-38029120: ACGCTTGAACCCTTGCTCTG.......TT......TT..................xxx...........A.---............-.............TTCTGCTGTGATTTTACATC
2R 40263414-40264380: ATTTTCTAACCACAGCAACG.......-...................A.......xxx........T....---......C.....TA.C......A...CAACGGGGAAGGATCGCTCC
2R 45193683-45192640: CAATTAATTTCCAACCATTT.......-.......A.....C......CT.....xxx............A---............-.............CATTCAACAGAAGATAAGCA
2R 52261880-52260739: ACTACTCTCCCTCGCCTTGT.......-..........A.........CT.....xxxG............---...........G-.............CGTGTATACATTAGCCATAT
X  11090120-11091791: TACTAGCCATCCTGCTATTG.......-.T....A...............A....xxx.T..G........T--.....A..G...-.A...........CATTGTGCGCTTGAACCAGG
X  10428461-10430292: ACCACCGCCAGCCCCCAAGG.......-.....TG..G.................xxx........GG...TTT..G....AAG.A-.A...........CCAGCAATGGGGTAGCCTGC
X   6203512-6205032 : CACGAAAGGGGCACTCGGTG..T....-........T.A.AT.C.......T...xxx.......G..T.TT--.......A.C.A-.............CGGTGGTACACTTTGCACCC
```

Legend to Supplementary Figure S4c. Alignment of mosquito *TransibN3_AG* elements to the *TransibN3_AG* consensus sequence. The consensus sequence is shown in the top line. Dots indicate nucleotide identity with the consensus sequence; hyphens represent alignment gaps. The internal portion of *TransibN3_AG* is not shown and is marked by xxx. Target site duplications are highlighted. Coordinates of the reported elements are shown in the first two columns (sequence ID, beginning-end).

Figure S4d.

```
TransibN1_DP               :                    CACTATGGTCCGAACGACCACTTTTGTTGGCCAAAA-CCTAxxxTTAATTTTGGCCAACAAAAGTGGTCGTTCGGACCATAGTG
Contig1006 3582196-3583547: CTCTTTCTTTTGGGCCATTG....................................-....xxx........................................CATTGTAGCATGACAATAAA
Contig1160 1464143-1462912: ACACTACCGTACATACACCC....................................-....xxx........................................CACCCCATGGACACACACTC
Contig138   431334-430521 : CATAAAAGATGCGTGCAATG....................................-....xxx........................................CCAATGGTTAGAGAGCGAAA
Contig1421  431059-429882 : GGTGCTGAGGCGAGGCGAGG....................................-....xxx........................................CGAGGGCTGGAGCACGTAGC
Contig1828  716750-717172 : AACGACGCCGCCGGTCGCCG............C....-..AA..............-.AA.xxx........................................CATGGTGCGCCGGCAGCAGC
Contig2138    3719-4888   : ATATTTTTGTCGAAACATTG..................................C.-....xxx...................AC..................ACATTGTTTGCTGGGATATTG
Contig2803  345220-344414 : AGGCAAAAATTCCGCCGCTG..........................C.........-....xxx..........G.............................CGCTGTCGCCAAGCTGCGTT
Contig3323 1183750-1182915: GGCTATTTCTCTTTACAGTG.............................A......A....xxx.........A....................T........CCAGTGACGTGACTCCCGCCG
Contig4055  469237-470472 : ACACATTCGGAGAGACAGTG....................................-....xxx.................G......................CAGTGTAGCCACCGCAAAGG
Contig410   329647-332793 : TGAACTCCTCCCTCCCTCCG..............T.....................-....xxx.........................A..............CTCCGTCCTGACCGATGAAT
Contig4374  876591-877958 : TGGAGGCACTGAAGGCACTG....................................-....xxx........................................CACTGCCACGGGACTCTGTG
Contig4922 2719081-2717796: GATCCGCAAGCGAGGGGTTG....................................-....xxx........................................GGTTGGTTGCATTTGAAGGT
Contig5482  238752-239176 : GACTGCACACGCCACCACCC...........C........................-...Txxx.A.T........................G...........CACCCCCTCGCGAGCACCGA
Contig6780  952419-953428 : CGAAAAGGCCGGCCATGATG....................................-....xxx........................................TGATGGTTCGCTAACTCTTC
Contig7446 1535599-1534365: CTCTCTCTTTCCTCACGGTG....................................-....xxx........................................CGGTGGTCTTTGCGGGTCCT
```

Legend to Supplementary Figure S4d. Alignment of fruit fly *TransibN1_DP* elements to the *TransibN1_DP* consensus sequence. The consensus sequence is shown in the top line. Dots indicate nucleotide identity with the consensus sequence; hyphens represent alignment gaps. The internal portion of *TransibN1_DP* is not shown and is marked by xxx. Target site duplications are highlighted. Coordinates of the reported elements are shown in the first two columns (sequence ID, beginning-end).

Figure S4e.

```
Hopper                       :                 CACTATGGGGCATTTGGCCTGTTTTTTTT---ACAAAAATTAAxxxCACATTATTGT--AAAAAAAACAGGCCAAATGCCCCATAGTG
arm_X        3321783-3320398 :TAAGCCAAATTTACAATGCG.............................---...........xxx......T....-A.............................TAATGGTCCACATGTGCAGT
arm_X        7692469-7690997 :TTTTTAATTTTATACCGCTA.............................---...........xxx......T....--...........................C.CGCTAGGCTGCAGCTAGCTG
arm_X       11113816-1111529 :GTTGACGGGCCGTGCCATTG.............................---...........xxx...........--.............................CATTGTGGCGACCTTAATGG
arm_X      21065414-21065981:AAAATATGGACCTAATTAATG.............................T--...........xxx...........AA.............................GCAATGCATGAACAATACGT
arm_3R     14159016-14157541:GATCGAAAATAAAGACATTG.............................---...........xxx...........--.............................CATTGGTGAGGTGTTGAAAT
arm_3R     23683981-23682517:GGTCGCGTATTGGACCAATG.............................---...........xxx...........--.............................CAATGCTAATCTGACGGCGG
arm_2R       2651771-2653244 :ATTTTCGTGTGGCGCCGCTG.............................---...........xxx...........--...................T.........CGCTGTCTGCGTGGGCCTCA
arm_2R        818021-816534  :AGAGCCTGGAGCCACCTCTA.............................T--...........xxx...........--.............................CTCTAGAGCCACATCGAATG
arm_X      21643270-21644741:TGCATAGTGCCAAACCAATG.............................---...........xxx...........--.............................CAATGTATGGCCGTTACGCA
arm_2L     11615564-11614088:ACCAAAGCCACGCCCCAATG.............................---...........xxx...........--.............................CAATGCCCCCCAGCCATCCA
arm_2L     14466034-14467508:CTGGATGCGTTGCCCCACAG.............................---...........xxx...........--.............................CACAGCTCGGAGCCATAAGT
arm_3L     23114503-23113036:CAAAAATTCGAGAGAGCCTG.............................TT-...........xxx...........--.............................GCCTGGAGCCACCTCTAGAG
3h            101990-100832  :CCCAGCAACTCCGCACAATG..T.........................CT--...........xxx...........--.............................CAATGTTGCTAATCTTCCGC
3h            537419-538842  :AAGAGGCGGATGGGGCAGTG.............................TTT...........xxx...........--.............................CAGTGGTCTGATCACGGGGA
AABU01002545    8291-9763    :TGTAACCAATCACACCATTC.............................T--...........xxx...........-A.............................CATTCGTGAAATTCCTTTAA
AABU01002746 156961-15550     :CTGTCCTCTTCGGCACTATG.............................TT-...........xxx...........--.............................CTATGTTGTGTAGATTCACC
AABU01002753  12062-12884    :GTATTGTCAAATGGACAACG.............................---...........xxx...........---..................A.........CAACGGTACCACCAAAGACA
```

Legend to Supplementary Figure S4e. Alignment of fruit fly *Hopper* elements to the *Hopper* consensus sequence. The consensus sequence is shown in the top line. Dots indicate nucleotide identity with the consensus sequence; hyphens represent alignment gaps. The internal portion of *Hopper* is not shown and is marked by xxx. Target site duplications are highlighted. Coordinates of the reported elements are shown in the first two columns (sequence ID, beginning-end).

Figure S4f.

```
TransibN1_DM                 :                    CACTATGGGAAATTTGACCACTTTTTCTGGCCAAAA-TTAATxxxTAGGTTTTGGCCAAAAAAA---GGGGGTAAATTTCC-CATAGTG
arm_2L 21096809-21096313 :TGCTCCATGTTATCAGACTG.................A..................-....Axxx...................---..............-......CGACTGGCGACAAGAAATATG
arm_2L 21318542-21317319 :-GGCAGGGGCACCCACAAGGG..............T.............A......-.G...xxx.G.C....A....G.....---.T............-.......CAAGGGTCTAAGTGAGAGGC
arm_3L 21700662-21700244 :CTATGCACACGCAACCACCA...................................T-.....xxx.........T.......-----A.............A.......CACCCTACCGCAAACACACA
arm_3L 21812579-21813116 :TTCGCTCACTCTTCCTCTTG...............T.......C......T.....-.G...xxx...................A--..............-.......CATTGGAACGAGCCATCGGA
arm_X   1939342-1938788  :CACACACACATGGACCGCGG.........C.GC.C..T..-...........C...-.....xxx.G.....A...A.......AA-.......C......-.......CGCTGGTGTGCACGCGATTA
arm_X  15386725-15387583 :CGATGTTCCCTTCGCCGGCG..............................A.....-.....xxx.TT...............----.A............-.......CGGCGGTTCGGTCAAAGAAG
arm_X  21998594-21999112 :ACATTTGCGCCTGAGCACCG.............C.ACGT.................-.....xxx.T..A........T.....---.T......T.....-A......CACCGACCAGCCGGCCTATA
arm_X  22141281-22140789 :ACACACGCATAGAGACGTTG.......................G............A.....xxx.G.T..............-GGGCCAACA.T......-.......CGTTGATGTGCTCCCTCGTG
```

Legend to Supplementary Figure S4f. Alignment of fruit fly *TransibN1_DM* elements to the *TransibN1_DM* consensus sequence. The consensus sequence is shown in the top line. Dots indicate nucleotide identity with the consensus sequence; hyphens represent alignment gaps. The internal portion of *TransibN1_DM* is not shown and is marked by xxx. Target site duplications are highlighted. Coordinates of the reported elements are shown in the first two columns (sequence ID, beginning-end).

Figure S4g

```
Transib-N1_SP                            CACAGTGGGCCAGGGAGGAACCAAAGTGCGCCAAAAxxxTTTTGGCGCACTTTGGTTCCTCCCTGGCCCACTGTG
Contig12895 5655-6545:ATGGACCGGTGGACCGGCGCACAGTGGGCCAAGGAGGAACCAAAGTGCGCCAAAAxxxTTTTGGCGCACTTTGGTT-CTTCCTGGCCCACTGTGCGGCGGTCGAATAGTTAAAA
Contig23311  409-1274:TAAGCTCTACGCAGGCGCCGCACAGTGGGCCAGAGAGGAACCAAAGTGCGCCAAAAxxxTTTTGGCGCACTTTGG-TCCACCCTGGCCCACTGTGCGACGGCTTTCAGTCCTCTT
Contig29183 3334-3891:CAATTAACCAGGGACCGACGCACAGTGGGCCAGGGAGGAACCAAAGTGCGCCAAAAxxxTTTTGGCGCACTTTGGTTCCTCCCTGGCCCACTGTGCGACGGCCCCCCGACGGCCA
Contig30324 6923-6008:AAGAAAGTCAGCGAGCGGCGCACAGTGGGCCAGGGAGGAACCAAAGTGCGCCAAAAxxxTTTTGGCGCACTTTGGTTCCTCCCTGGCCCACTGTGGAGCGTAGCGAGCGAGCTTT
Contig36576 2448-3366:TCGGGAGCGCACTGCCGCCGCACAGTGGGCCAGGGAGGAACCAAAGTGCGCCAAAAxxxTTTTGGCGCACTTTGGTTCCTCCCTGGCCCACTGTGCGCCGCCGCCGCCGTGGCAA
Contig51741 1417-2323:GGAAGTACGTTCCTGCGACGCACAGTGGGCCAGGGAGGAACCAAAGTGCGCCAAAAxxxTTTTGGCGCACTTTGGTTCCTCCCTGGCCCACTGTGCGACGGTTCACGATTACGAA
Contig58992  635-1554:TTCGCGTAACCATGGCAACGCACAGTGGGCCAGGGAGGAACCAAAGTGCGCCAAAAxxxTTTTGGCACACTTTGGTTCCTCCCTGGCCCACTGTGCGCCGGGGCGTACTGATGGG
Contig59342 3343-2426:CCAACTTCATGTTCACGCCGCACAGTGGGCCAGGGAGGAACCAAAGTGCGCCAAAAxxxTTTTGGCGCACTTTGGTTCCTCCCTGGCCCACTGTGCGCCGGGGCACCGAGCGTAT
Contig6151  1755-853 :ACCCCGGGCCAGGGGCGGCGCACAGTGGGCCAGGGAGGAACCAAAGTGCGCCAAAAxxxTTTTGGCGCACTTTGGTTCCTCCCTGGCCCACTGTGCGGCGGAGCGTCATCCAAAA
Contig61636  561-1552:GTCTGTATCGGGGGCCGGCGCACAGTGGGCCAGGGAGGAACCAAAGTGCGCCAAAAxxxTTTTGGCGCACTTTGGTTCCTCCCTGGCCCACTGTGCGGCGGTCTCATGGGCTTTC
Contig68912   65-978 :ATTGGCATCAAGCACCGACGCACAGTGGGCCAGGGAGGAACCAAAGTGTGCCAAAAxxxTTTTGGCGCACTTTGGTTCCTCCCTGGCCCACTGTGCGACGATCAGTCGATCAAAT
```

Legend to Supplementary Figure S4g. Alignment of sea urchin *TransibN1_SP* elements to the *TransibN1_SP* consensus sequence. The consensus sequence is shown in the top line. The internal portion of *TransibN1_SP* is not shown and is marked by xxx. Target site duplications are highlighted. Coordinates of the reported elements are shown in the first two columns (sequence ID, beginning-end).
