# Supplementary material for: RAG1 Core and V(D)J Recombination Signal Sequences Were Derived from Transib Transposons
Source: PLoS Biol. 2005 May 24;3(6):e181. doi: 10.1371/journal.pbio.0030181 (PMC1131882; doi:10.1371/journal.pbio.0030181)
Supplement: Supplementary file 2 [file pbio.0030181.st001.doc]

| Species | | GenBank  sections | E-values |
| --- | --- | --- | --- |
| Common name | Latin name |  |  |
| Fruit Fly | Drosophila melanogaster | nr, wgs, est, tr | 10^-103^-10^-5^ |
| Fruit Fly | *Drosophila pseudoobscura* | htgs, wgs, tr | 10^-108^-10^-5^ |
| Fruit Fly | *Drosophila willistoni* | tr | 10^-75^-10^-5^ |
| Fruit Fly | *Drosophila yakuba* | wgs, tr | 10^-102^-10^-5^ |
| Fruit Fly | *Drosophila ananassae* | tr | 10^-65^-10^-5^ |
| Fruit Fly | *Drosophila mojavensis* | tr | 10^-60^-10^-5^ |
| Fruit Fly | *Drosophila simulans* | tr | 10^-60^-10^-5^ |
| Fruit Fly | *Drosophila erecta* | tr | 10^-49^-10^-5^ |
| Fruit Fly | *Drosophila auraria* | gss | 10^-34^-10^-5^ |
| Yellow Fever Mosquito | *Aedes aegypti* | htgs, wgs, gss, tr | 10^-87^-10^-5^ |
| African Malaria Mosquito | *Anopheles gambiae* | wgs, tr | 10^-82^-10^-5^ |
| Silkworm/Silk Moth | *Bombyx mori* | wgs, est | 10^-58^-10^-5^ |
| **Red Flour Beetle** | *Tribolium castaneum* | tr | 10^-55^-10^-5^ |
| Dog Hookworm | Ancylostoma caninum | gss | 10^-42^-10^-5^ |
| Freshwater Flatworm | Schmidtea mediterranea | tr | 10^-11^-10^-5^ |
| Soybean Rust | *Phakopsora pachyrhizi* | tr | 10^-47^-10^-5^ |
| Purple Sea Urchin | Strongylocentrotus purpuratus | tr | 10^-173^-10^-5^ |
| Hydra | Hydra magnipapillata | est, tr | 10^-77^-10^-5^ |
