# Supplementary material for: RAG1 Core and V(D)J Recombination Signal Sequences Were Derived from Transib Transposons
Source: PLoS Biol. 2005 May 24;3(6):e181. doi: 10.1371/journal.pbio.0030181 (PMC1131882; doi:10.1371/journal.pbio.0030181)
Supplement: Supplementary file 3 [file pbio.0030181.st002.doc]

| **hAT family** | **gc content of the host genome**  **(%)** | **gc content of 35-bp insertion sites**  **(%)** | **gc content of 15-bp insertion sites**  **(%)** | **gc content of 8-bp target site duplications (%)** | **number of analyzed elements** |
| --- | --- | --- | --- | --- | --- |
| Pegasus^1)^ | 44 | 45 | 44 | 51 | 34 |
| HATN4_AG^2)^ | 44 | 47 | 52 | 45 | 14 |
| HAT2N_AG^2)^ | 44 | 49 | 48 | 47 | 12 |
| Hobo^3)^ | 42 | 38 | 38 | 41 | 20 |
| hAT-1N_DP^4)^ | 46 | 41 | 46 | 40 | 19 |
| hAT-1N_SP^5)^ | 37 | 30 | 31 | 27 | 41 |
| HAT1N_DR^6)^ | 36 | 44 | 44 | 36 | 110 |
| HAT-2N1_DR^6)^ | 36 | 35 | 33 | 39 | 47 |
| HAT-N19_DR^6)^ | 36 | 34 | 33 | 28 | 41 |
| Average |  | 40 | 41 | 39 |  |
